# Classification of apatite structures via topological data analysis: a framework for a ‘Materials Barcode’ representation of structure maps

**DOI:** 10.1038/s41598-021-90070-4

**Published:** 2021-06-02

**Authors:** Scott Broderick, Ruhil Dongol, Tianmu Zhang, Krishna Rajan

**Affiliations:** Department of Materials Design and Innovation, University at Buffalo, 120 Bonner Hall, Buffalo, NY 14260-5030 USA

**Keywords:** Computational methods, Materials for energy and catalysis

## Abstract

This paper introduces the use of topological data analysis (TDA) as an unsupervised machine learning tool to uncover classification criteria in complex inorganic crystal chemistries. Using the apatite chemistry as a template, we track through the use of persistent homology the topological connectivity of input crystal chemistry descriptors on defining similarity between different stoichiometries of apatites. It is shown that TDA automatically identifies a hierarchical classification scheme within apatites based on the commonality of the number of discrete coordination polyhedra that constitute the structural building units common among the compounds. This information is presented in the form of a visualization scheme of a barcode of homology classifications, where the persistence of similarity between compounds is tracked. Unlike traditional perspectives of structure maps, this new “Materials Barcode” schema serves as an automated exploratory machine learning tool that can uncover structural associations from crystal chemistry databases, as well as to achieve a more nuanced insight into what defines similarity among homologous compounds.

## Introduction

One of the classic questions in materials crystal chemistry is: why do atoms arrange themselves in the way they do? To address this challenge, the concept of phase homologies has been widely used. Homologous series of compounds are those that seem chemically diverse but appear capable of producing each chemical member in a stoichiometric relationship in a unique crystal structure “type”. In this context, the use of classification maps that partition different genres of chemistry for different structural arrangements is a foundational tool in chemical crystallography and the evolution of structure maps, especially in inorganic structures. Structure mapping has played an important role as an a priori guide for finding stable phases ^1^ and serving as a visualization tool for structure–property relationships in a bivariate way. Physical factors governing stable crystal structures serve as the coordinates of structure maps.

After physical factors are carefully chosen, each compound can be spatially identified by its structure type. From an informatics perspective, a structure map is a classification tool whereby, through the choice of appropriate coordinates, one can map clustering of crystal-structure-related data. There is, of course, a long and rich history of such maps including Mooser–Pearson plots ^2^, Philips and van Vechten diagrams ^3,4^ Goldschmidt diagrams ^5^ and Pettifor plots ^1^, just to mention a few examples. Each of these mapping schemes identifies some key parameters related to electronic or crystal structure information which is usually placed on orthogonal axes and the occurrence of a given crystal chemistry is then plotted accordingly. The resulting diagram maps out the relative position of structure types from which one tries to discern qualitatively if there are strong associations of certain crystal types to certain bivariate combinations of parameters. The key of course in developing or selecting the appropriate framework of descriptors or combinations of descriptors is to capture both the key bonding and crystallographic metrics that would permit classification. Rather than relying solely on heuristics or phenomenological observations, our group and others have utilized data driven methods to create the framework for creating structure maps ^6–8^. This process then extracts the dominant metrics among the attributes.

We provide an alternate data driven approach of objectively classifying datasets using concepts from algebraic topology, namely, persistent homology ^9–15^. Topology is inherently a classification system that deals with qualitative geometric information and we propose to demonstrate in this study the value of using such a machine learning approach to uncover fundamental insights into the role of coordination environments in complex crystal chemistries in a completely unsupervised learning process. The objective of this work is to demonstrate how by exploring different machine learning methods we can gain insight into the underlying features of apatite crystal chemistry classification, which is a topic of great study especially in the field of chemical crystallography and geochemistry (where apatite structures are most discussed). Our work shows how machine learning methods provide another lever in the toolkit of interpretation that has traditionally used heuristic and phenomenological analysis to generate hypotheses on classification criteria in complex chemical systems.

## Computational details

### Data input

For this paper, we will build upon our earlier work on the apatite family of crystal structures ^16^. The apatite crystal structure has the general formula of A_5_(BO_4_)_3_X, with A representing crystallographic sites typically accommodating large cations, B accommodating smaller cations, and X occupied by anions. From this structure, we can identify a series of descriptors related to size effects, bond lengths, crystallographic angles and charge effects (Table 1). As the input descriptors have differing units, the data was first normalized so that the data is not sensitive to the unit chosen. The data was first mean centered and then the values were divided by the standard deviation for each descriptor. This provides data in the same format as our prior work so that we can compare and contrast the results from each. In that prior work, we identified classifiers based on specific bond angular distortions through Principal Component Analysis (PCA) to identify a pair of the strongest variables of importance. They were found to be useful to see classifications based on the stoichiometry of site occupancy in the apatite family of structures. However, the clustering boundaries did not necessarily work for all of the compounds studied, and in this paper we apply TDA to relax the objective of identifying just a few of the dominant descriptors. The ‘big data’ perspective of this work refers to the high dimensionality of the data set and not only the number of samples. When coupled with the descriptor data set for each sample and inherent uncertainties in knowing all the complexities associated with chemical bonding, TDA serves as a useful exploratory unsupervised machine learning approach to explore connectivity.

**Table 1:** Descriptor set for apatite systems, with the corresponding descriptions.

	Descriptor	Description
1	a	Lattice constant
2	c	Lattice constant
3	c/a	variable axial ratio
4	r_AI_	Shannon's ionic radii of A^I^-site ion
5	r_AII_	Shannon's ionic radii of A^II^ site
6	r_B_	Shannon's ionic radii of B-site ion
7	r_X_	Shannon's ionic radii of X-site ion
8	Av CR	Average crystal radius
9	A_EN_–O_EN_	Electronegativity difference between A and O atoms
10	B_EN_–O_EN_	Electronegativity difference between B and O atoms
11	A_EN_–X_EN_	Electronegativity difference between A and X atoms
12	A_EN_–B_EN_	Electronegativity difference between A and B atoms
13	ψ_AI–O_	Bond angle of A^I^–O1 bond
14	ψ_AI–O_^(Z=0)^	Bond angle of A^I^–O1 bond for z = 0 at A^I^
15	δ_AI_	Counter-rotation angle of A^I^O_6_ unit
16	ϕ_AI_	Metaprism twist angle
17	α_AI_	Orientation of A^I^O_6_ unit
18	< τ_O–B–O_ >	Average O–B–O bond-bending angle
19	ρ_AII_	A^II^–A^II^ triangular side length
20	α_AII_	Orientation of A^II^–A^II^–A^II^ triangle
21	ϕ_O3–AII–O3_	O3–A^II^–O3 angle
22	A^I^–O1	Distance between A^I^ and O1 atoms
23	A^I^–O1 ^(z=0)^	Distance between A^I^ and O1 atoms, for z = 0 at A^I^
24	Δ_AI–O_	Difference in A^I^–O1 and A^I^–O2 bond lengths
25	Δ_AI–O_^(z=0)^	Difference in A^I^–O1 and A^I^–O2 bond lengths for z = 0
26	< B–O >	Average B–O bond length
27	A^II^–X	Distance between A^II^ and X atom
28	A^II^–O3	Distance between A^II^ and O3 atom
29	E_total_	Total energy from DFT

We have defined a descriptor set encompassing atoms, bonds, and crystallographic measures of the apatite structure. The descriptors are from the geometrical parameterization scheme of Mercier et al. ^18,19^, as well as descriptors from Shannon ^20^ and Pauling ^21^. In that work, they quantified the bond distortions based on algebraically independent bond lengths and angles. From this data, we want to track the relationship between geometrical parameters and structure.

Using persistent homology, algorithmically we can rapidly explore all permutations and combinations of the connectivity of the descriptors in the context of algebraic topology. Of course, the results of any descriptor-based input are going to be influenced by the nature of the descriptors. Hence, we are using two different types of data dimensionality reduction methods using the same descriptor library. The PCA based analysis ^16^ has provided a physically interpretable analysis (e.g., site occupancy clustering of most of the compounds). Even when outliers were found, subsequent analysis using DFT calculations provided an explanation of why those outliers existed in that PCA projection ^17^, demonstrating how strong classifiers are of value not only to identify global patterns but also to identify meaningful outliers. Hence, for this study we are utilizing a descriptor space that has demonstrated its value in physically interpretable analyses.

**Figure 1:**
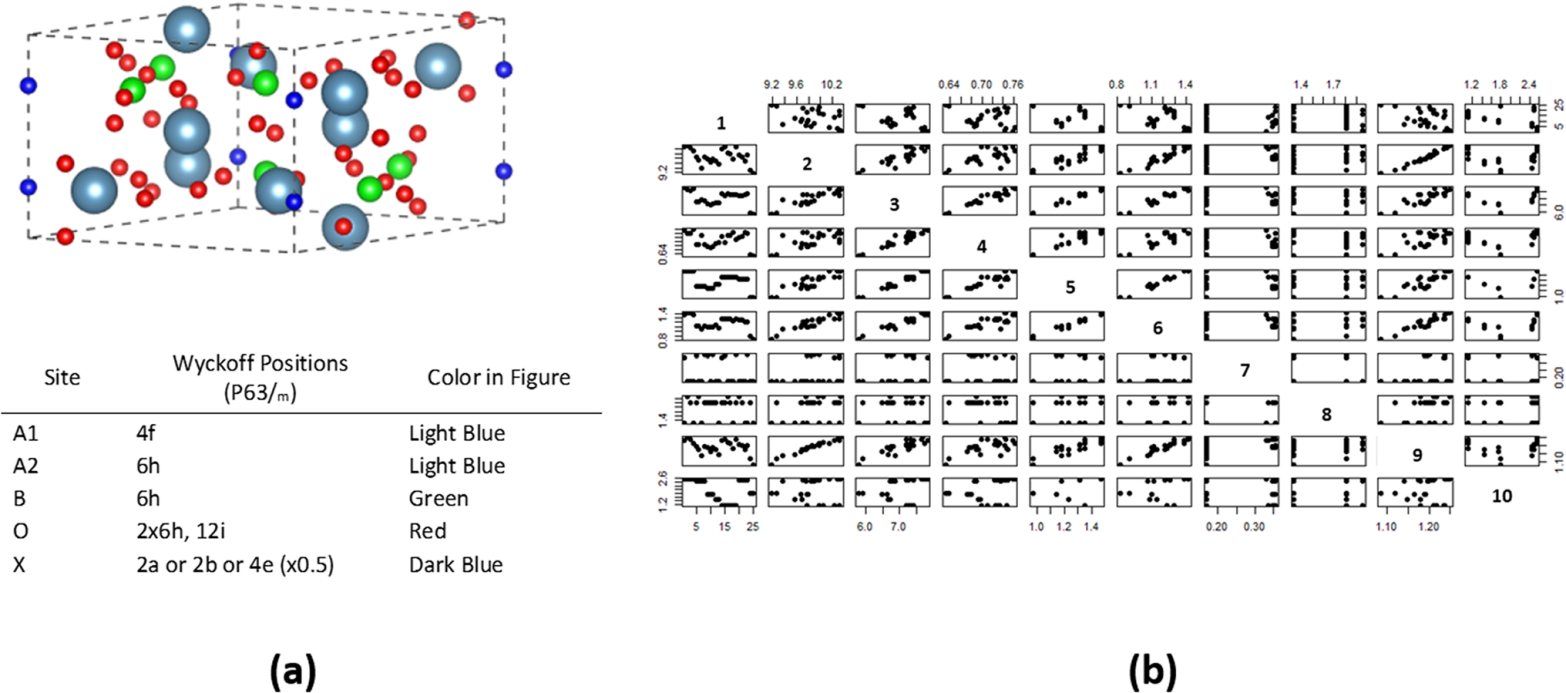
(**a**) The building of the apatite structure (A_5_(BO_4_)_3_X) starts with the Wyckoff positions. (**b**) Standard structure map representation for defining correlations. For clarity, only the relationships of the first 10 descriptors (correlating with the labels in Table 1) are shown.

The apatite structure is shown in Fig. 1, as well as the traditional structure map plotting of the descriptors. In total for the 29 descriptors, there are 406 total pair-wise structure maps. In this paper, we compress these various correlations into a single 2D representation, based on the combined correlations of the descriptors. The challenge in this is that the strong intercorrelations in the data make the extraction of any meaningful design rule challenging. The general notion of materials similarity may have many facets, and one structure map is not enough to contain all of the facets. There has been a series of crystallographic parameters which have been proposed to govern the apatite character and behavior (Table 2). Therefore, a breadth of knowledge and data exists in the case of apatites, but a unified similarity metric capturing all of these constraints does not exist. In this paper, a clear classification is developed through the barcode representation, and comparisons with these prior works are discussed.

**Table 2:** Prior classifications of apatite or apatite-like (i.e. polysomatic apatites) compounds ^16,22–25^.

Descriptors	Outcome of analysis	References
Unit cell parameters	Structural classification of apatites	T.J. White and D. ZhiLi (2003)
Metaprism twist angle		
N in A_5N_B_3N_O_9N+6_X_Nδ_	Uncovering polysomatic apatites	T. Baikie et al.(2010)
Metaprism twist angle		
Distortion angle α_AII_	Classification of apatites—PCA derived selection of mapping descriptors : used for comparison here	P.V. Balachandran and K. Rajan (2012)
Distortion angle Ψ_AI–O1_		
A-site radius	Defining chemical search space	P. Ptáček (2016)
B–O Distance	Conductivity for a subset of apatites	M.S. Chambers et al. (2019)

These descriptors represent the crystallographic characteristics of the apatite which have been identified as most closely determining the property of the compound.

### Persistent homology

Persistent homology (PH) is the most commonly used method in TDA, which captures topological features in the high-dimensional data, such as connected components, loops, and holes, at multiple scales. All TDA methods treat high-dimension data as a point cloud ^26–31^. PH maps a set *X* of points in the high-dimensional space associated with a distance function. This mapping is modeled by placing a *ε*-radius disc centered at each point in X to form an overlapped space *τ*_*ε*_*(X)*. Here, *τ*_*ε*_*(X)* is defined as the set of all points in space within the distance *ε* from a certain point in *X*. A connection between two data points is established when the topological space occupied by their discs overlap. The number of connected components in *τ*_*ε*_*(X)* is equal to that of the points in *X* for a sufficiently small *ε*, while all of them combine into one component for a sufficiently large *ε*. Subsequently, the topological features for a given *ε*-radius captures information about the shape of data. It is natural to assume that different topological features are captured at different *ε*-radius. Hence, the fundamental idea behind PH is the study the evolution of the topological features in *τ*_*ε*_*(X)* as *ε* increases.

**Figure 2:**
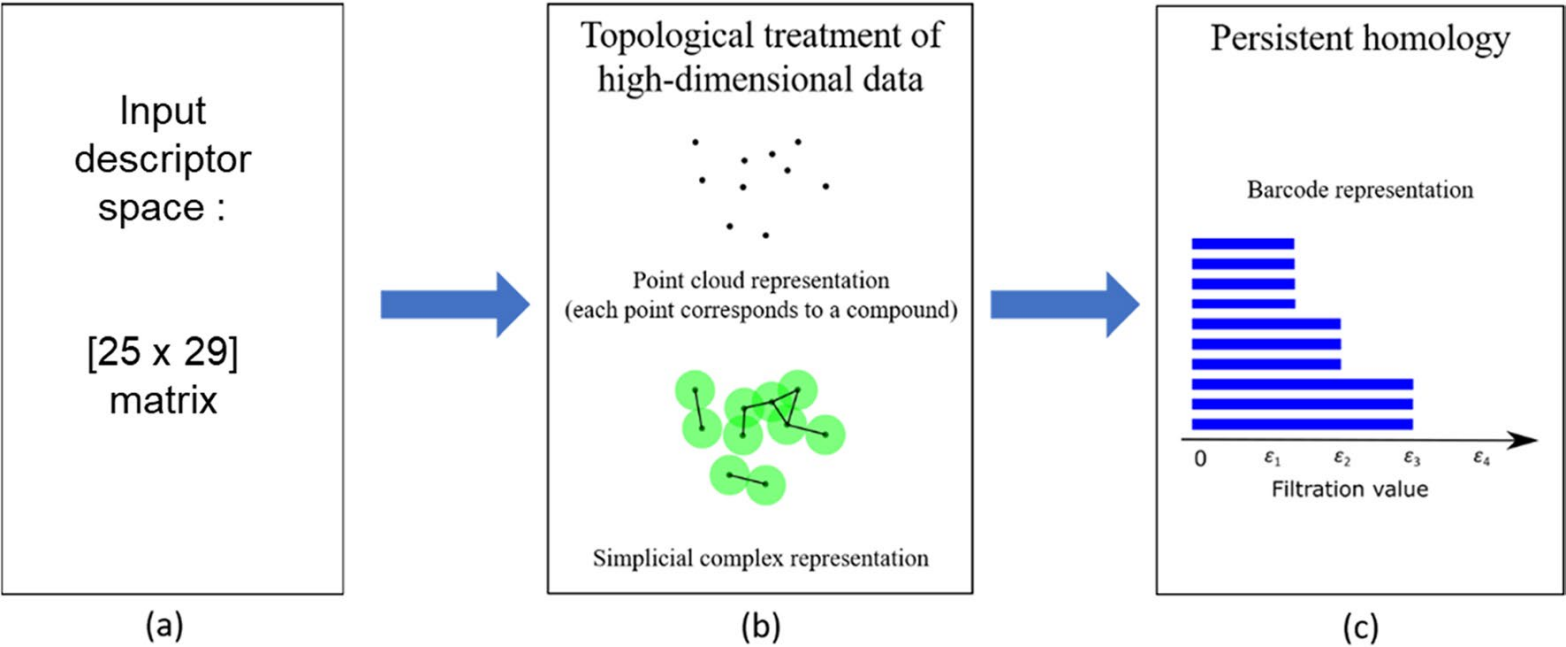
(**a**) The application of the TDA requires input descriptors, such as listed in Table 1, which creates, (**b**) the high-dimensional input space point cloud. Each data point in the point cloud representation of the descriptor space corresponds to an apatite compound. The ‘growing’ disc (highlighted by the green disc centered at each data point) results in formation of multiple topological features such as connected components. (**c**) The evolution of the topological feature is tracked using the barcode representation. The actual barcode for the apatite dataset is provided in Supplementary Materials.

In this work, we are primarily interested in the evolution of connectivity of apatite compounds in the descriptor space defined by Table 1. The geometrical descriptors of apatite compounds define the point cloud, Fig. 2a,b, where each point corresponds to an apatite compound. The point cloud incorporates the crystallographic and chemical bonding information. The evolution of connectivity of components is tracked through the overlap of ‘growing’ discs. In the computation routine, the evolution of *τ*_*ε*_*(X)* is modeled through a sequence of nested geometrical objects (simplicial complex), which is known as filtration. Here, we implement the Vietoris–Rips complex to define the connected components in the topological space. The evolution of the connected components can be visualized through a series of connectivity diagrams and compressed in static figure called a ‘barcode’, Fig. 2c. We provide the definition of connected components and Vietoris–Rips complex in the Supplementary Material.

### Interpreting the material barcode

**Figure 3:**
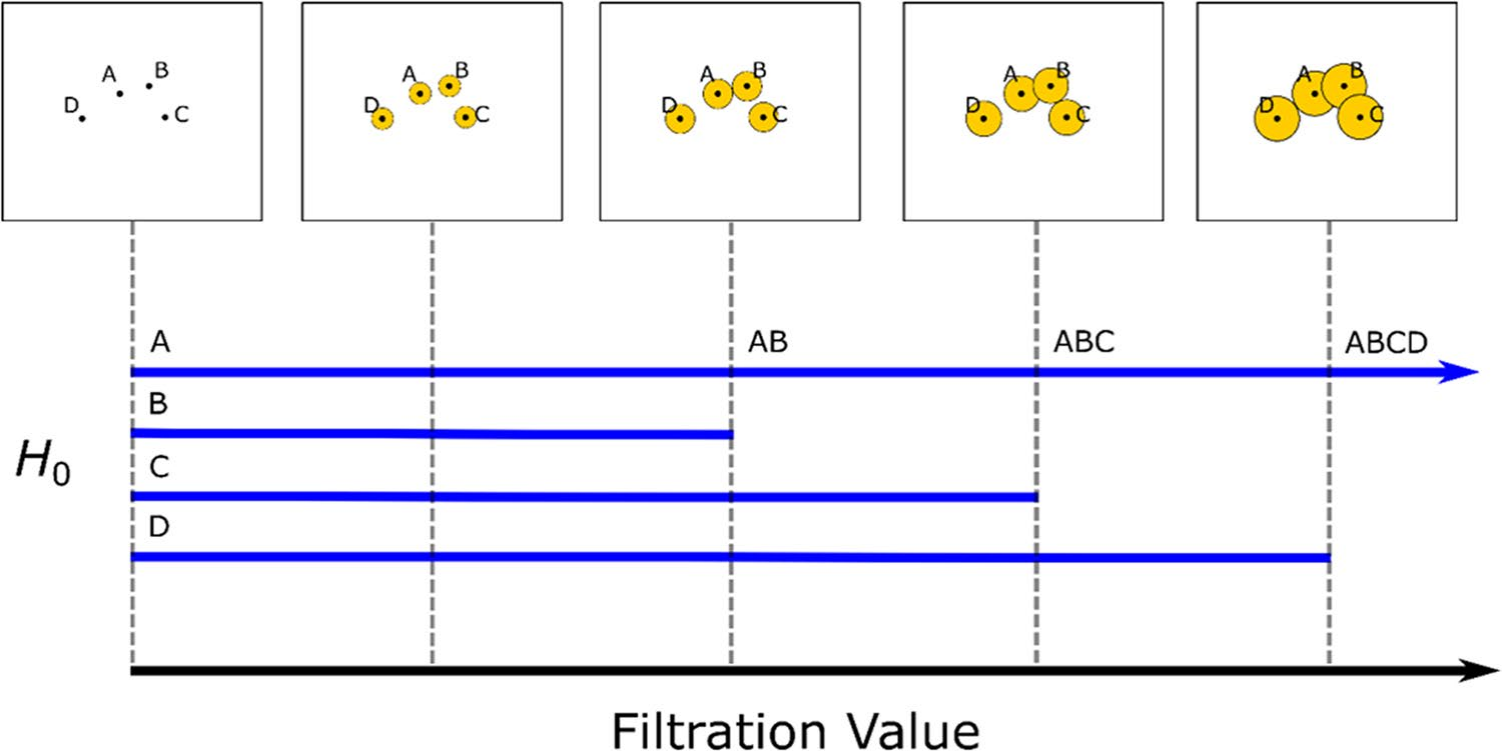
In this schematic representation of persistent homology, the topological space is defined by the four-data points (components) labeled A, B, C, and D in R^2^. The barcodes (solid lines) represent the lifetime of the components, whose length is determined by the filtration value. In this case, the filtration value is the radius of the ‘growing’ disc centered at each component. A connection between components is established at the contact point of two ‘growing’ disc. In the barcode, this event is represented by termination of a barcode for one component and simultaneous representation of the connected component by a single barcode. For example, in the third image, the discs of components A and B come in contact. This event is represented by termination of barcode for B, while simultaneously the barcode for A now represents the connected components AB until component C is connected. The arrow head indicates that the filtration value can increase to infinity.

While there are other approaches to represent similarity in high dimensional space ^32,33^, we are using the barcode as a visualization tool to readily see groupings of compounds. Figure 3 shows a schematic representation of the evolution of the thickening of the point cloud and corresponding barcodes on the four-data point (components) space labeled A, B, C, and D. In the 2D space, the thickening of the point cloud is represented by the ‘growing’ disc process centered at each data point. The filtration value is representative of the size (in 2D, radius) of the disc, while the arrow head indicates that the filtration value can increase to infinity. At any given filtration value, the topological space is the union of all the points in the ‘growing’ discs and each disc is a subset of this space.

From the algebraic topological perspective, the point-cloud itself is not interesting but the analysis of evolution of the resulting shape from thickening of the point cloud is of interest. In this work, we focus on the 0th-dimensional topological features of the PH (H_0_), which corresponds to the connectivity of the data points (i.e. connected components) in the finite parametric space represented by the barcode. The barcode encodes the evolution of topological features resulting from the thickening of the point cloud**.** It should be noted that we have added the results of mapping the H_1_ topological features in the Supplementary section. The classification results provide an assortment of patterns, when compared with the PCA and H_0_ topological feature maps. For instance, the bulk of the groupings appear to be most sensitive to the mapping of the phosphate compounds. However, other classifications include the detection of cluster of compounds that appear in the PCA analysis as part of a larger cluster of compounds. This does not detract from the focus of our main results but simply emphasizes how TDA combined with PCA in this case, serves as a powerful data exploratory tool.

Initially in Fig. 3, there are four-independent components with the radius of the disc being zero. Each disc grows independently. A connection between two components is established when two discs centered at different components come in contact. For example, in the third image of Fig. 3, components A and B form a ‘connected component’ as their discs’ connect. In the barcode illustration, this event is represented by the termination of the barcode for B, while simultaneously the barcode for A now represents the connected components AB until component C is connected. In PH, the onset of a bar is referred to as a ‘birth’ of a feature, while an end-point of the bar is referred to as the ‘death’ of the feature. In our case, all 0th-dimensional features of the components have ‘birth’ at zero filtration value. The ‘death’ of feature for B occurs at the third image, C at the fourth image, and D in the last image. The longest barcode is the most persistent (significant) feature of the point cloud, which in this case is the connected components.

In the finite parametric space, the radius of a ‘growing’ disc automatically tracks the dynamical aspect of the proximity of components in the form of a barcode such that length of the bar represents the non-static nature of the clustering of components. Therefore, the barcode conveys two important information; first, the classification of components based on the connected components at increasing filtration value. In such a case, it is natural to assume a stronger association between connected components at lower filtration values. Additionally, connected components at lower filtration values indicate the notion of higher degree of similarity based on the close proximity of components. For example, in Fig. 3, component A is far more likely to be similar to B than to D. Second, the identification of outliers (i.e., unique components) across potentially high-dimensional parametric space. In this work, we utilize the prior set of information to identify different classes of apatites from the barcodes.

In the application of PH in materials science domain, the finite parametric space is defined by the multiscale high-dimensional descriptor space. In this study, we use 29-dimenional input descriptor space, which provides a parametric framework for apatite structure and where each point in this space corresponds to a distinct apatite. The barcodes allow to define the connectivity of the apatites based on the 29-dimensional input space. More importantly, the barcode encodes similarity between apatites with high-degree of similarity associated with lower filtration values. As discussed earlier, different filtration values are used to classify apatites with lower values carrying higher importance, and therefore are a more critical factor for classification. Based on the concept of similarity, in this study we demonstrate how topological data analysis serves as an automated exploratory machine learning tool that can uncover structural associations from crystal chemistry databases, as well as to achieve a more nuanced insight into what defines similarity among homologous compounds.

## Results and discussion

Following the approach described, we generate a barcode using size, bonding, charge, and crystallographic descriptors. The barcode provides a framework to create a classification of compounds, when the classifiers do not have any obvious relationships. The similarity metric can be extracted from the barcode by taking snapshots at various filtration values. In a traditional 2D structure map, the closer two compounds are than the more similar they are. Generalizing this idea to the snapshots of the barcode, the sooner (i.e. lower filtration value) two compounds merge (to appear on the same bar), the higher similarity they have. In the extreme, we can think of it as two compounds with identical descriptors appear at the same point in a 2D structure map or share the same bar in a barcode no matter the filtration parameter. This then provides a framework for chemical substitution with minimal impact on properties, i.e., shorter bars indicate that the compounds on it have more similarity in their properties.

**Figure 4:**
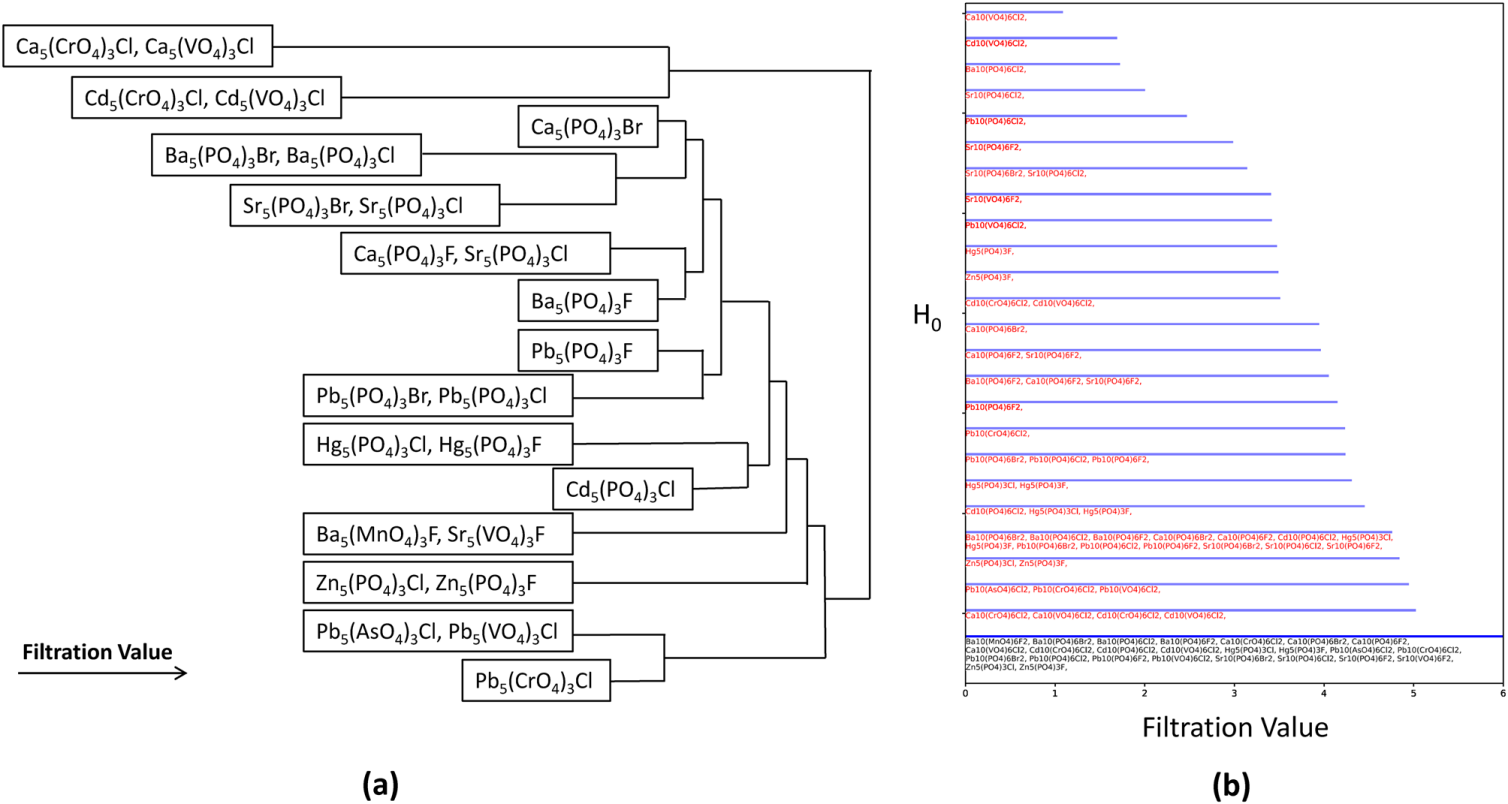
(**a**) The classifications extracted from the TDA derived barcode. As the filtration value increases, additional relationships picked up by the barcode appear. The similarity of the compounds decreases as the filtration value at which their relationship appears increases. This allows us to identify not only classifications at a given criteria but also the degree of similarity within those classifications. (**b**) The final barcode corresponding to this dendrogram.

Using this idea, we can then rank the clustering of compounds, and more importantly the ‘stability’ or persistence and existence of the influence of the input descriptors on the clustering of similarity of compounds. That is, when compounds merge together onto a bar at a low filtration value, that clustering persists until the final filtration value. We can then consider that to be a classification of high stability. The stability of the classification weakens as the initial merging occurs at larger filtration values. This dynamic aspect of the barcode (i.e. that it evolves with increasing filtration values) separates the approach from traditional clustering algorithms which provide only a single classification, with no stability ranking. To highlight the “dynamic” aspect of the barcode, a video of the evolution with increasing filtration value is provided in the Supplementary Material. From the series of barcodes for all filtration values (including the barcode shown at a single filtration value), we are able to come up with a ranking of the classification strengths (Fig. 4). The sooner the cluster forms corresponds with stronger classification or stability of the cluster. For example, the clustering of compounds with A = Ca or Cd, B = Cr or V, and X = Cl are the most strongly correlated, and the compounds with larger corresponding cluster numbers are more loosely similar. Conversely, Cd_5_(PO_4_)_3_Cl is the most unique compound, as it is the last compound which merges with any other compounds. The barcode results corresponding with this dendrogram are provided in the Supplementary Material.

The characterization of the complexities of the connectivity of descriptors defining the clustering of apatite compounds allows us to define potential chemical substitutions and targeted chemical design. The barcodes were defined using a descriptor set encompassing both size effects and crystallographic measures. Our prior work used a combination of PCA and k-means clustering to define the compounds with highest similarity. In that work, the similarity was defined based on A^I^–O1 twist angle and orientation of A^II^ bond units. This approach followed a traditional structure map approach where the key descriptors are identified and serve as the axes, in order to make the relationships more easily visualized. However, the TDA approach provides an added level of information by exploring the strength and evolution of the correlations. That is, at each filtration value we identify correlations and these correlations expand as we increase the filtration value. Therefore, this provides a dynamic representation of a structure map. The traditional structure map provides a representation of the data with the assignment of correlations requiring some definition of thresholds. However, in this work, the degree of correlation is ranked based on the filtration value at which it appears, and therefore this provides a dynamic representation of the stability of correlations.

**Figure 5:**
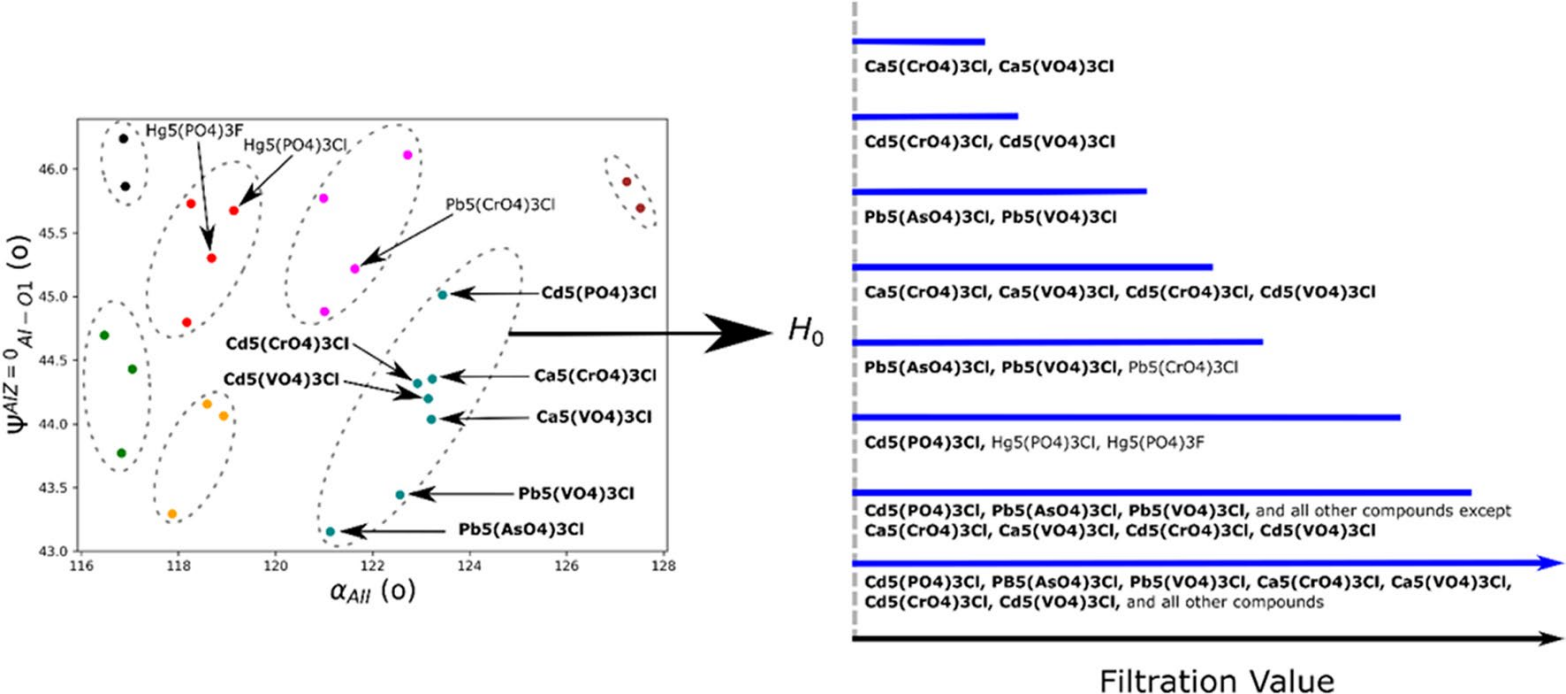
Comparison of the similarities resulting from (left) the combined PCA + k-means approach and (right) the TDA approach. For demonstration purposes, we focus on one cluster, and have chosen the cluster which contained the most points. A plot with all points labeled is provided in the Supplementary Material. From this, we have a ranking of the classification strength, while also identifying some differences (such as Ca_5_(PO_4_)_3_Cl, Pb_5_(AsO_4_)_3_Cl and Pb_5_(VO_4_)_3_Cl having little similarity with the other compounds in the cluster). The further interpretation of this plot, as well as for all of the other clusters, is laid out in Table 3.

Figure 5 shows the different representations, with the barcode evolution shown for the cluster which contained the most compounds. In this case, the structure map ranks all 7 compounds as having similarity without any further assessment. This shows how TDA provides different levels of ranking, with the bars terminating at lower filtration values having higher similarity.

Table 3 then compares the results and the interpretation resulting from all seven clusters labeled in the structure map, with the first row providing the interpretation resulting from Fig. 4. The results summarized in Table 3 are obtained by integrating the results from the PCA and TDA. As noted earlier, the PCA studies showed the dominance of X-site occupancy on clustering while also identifying outlier clusters that were governed by other criteria. The TDA results appear to capture a clustering pattern that seems to be based on coordination number. We then compared the clustering of compounds that TDA showed which do not fit with the k-means clustering pattern in the PCA results. These differences directed us as to which features might be common in those compounds that appeared clustered. This type of systematic exploration led to the descriptions provided in Table 3. We used the PCA derived structure map as a starting point as the clustering patterns are based on a physically interpretable descriptor space. The physical interpretation of the clustering in the TDA barcode is not so straightforward and hence by leveraging both data dimensionality reduction methods, we are able to get a more detailed and granular understanding of the design rules underlying the classification of apatites.

**Table 3:** A summary of some of the key new genres of crystal chemistry metrics uncovered by TDA to provide a higher level of granularity and more insight to define similarity between different apatite chemistries.

Clusters defined from PCA + k-means structure maps ^16^	Comparison to clustering from TDA barcode structure map—present study
Cd_5_(PO_4_)_3_Cl, Ca_5_(CrO_4_)_3_Cl, Cd_5_(CrO_4_)_3_Cl, Cd_5_(VO_4_)_3_Cl, Ca_5_(VO_4_)_3_Cl, Pb_5_(VO_4_)_3_Cl, Pb_5_(AsO_4_)_3_Cl	(1) Ca_5_(Cr,VO_4_)_3_Cl, Cd_5_(Cr,VO_4_)_3_Cl, and Pb_5_(As,VO_4_)_3_Cl are all identified as having high similarity even with changing B-site atoms. The similarity in each case is due to the common A(II)–O and A(II)–X bonds. These bonds define the edge of a polyhedron with CN = 7. (2) Cd_5_(PO_4_)_3_Cl has some similarity with the Pb compounds, representing the impact of the A(I)–O bonds. The A(I)–O bonds form a polyhedra with CN = 9, and therefore CN = 9 polyhedra have lower impact on defining similarity than CN = 7
Ba_5_(PO_4_)_3_F, Ba_5_(MnO_4_)_3_F, Sr_5_(PO_4_)_3_F	This cluster captures the B–O bond in the crystal. The B–O bonds form a polyhedra of CN = 4. The similarity in these compounds is low compared to the above cluster. This is due to the CN = 4 polyhedra having the least impact in classifying similar chemistries. Ba_5_(MnO_4_)_3_F has particularly little similarity with the other compounds although A(II)–X bond is the same. This likely reflects increased distortion in the bond angles caused by Mn. The twist angles used in the prior study do not take B–O bond angles into account
Sr_5_(VO_4_)_3_F, Pb_5_(PO_4_)_3_F, Ca_5_(PO_4_)_3_F	These compounds are not identified as being similar. This is due to having different A–O and A(II)–X bonds, and therefore there is no similarities in the two dominant coordination polyhedra
Ba_5_(PO_4_)_3_Br, Ba_5_(PO_4_)_3_Cl	These compounds are ranked as having high similarity. This is reflected through the combination of both A(I)–O and B–O bonds. While this does not include the A(II) atoms which corresponds with the main classifier, these compounds do share two coordination polyhedra
Sr_5_(PO_4_)_3_Br, Hg_5_(PO_4_)_3_Cl, Hg_5_(PO_4_)_3_F, Sr_5_(PO_4_)_3_Cl	Sr_5_(PO_4_)_3_X are similar and Hg_5_(PO_4_)_3_X are similar, but they have low similarity between each other. The similarity is due to the common A(I)–O and B–O bonds, thus representing two coordination polyhedra. The Sr and Hg compounds are dissimilar with each other due to only having common B–O bond, which has the least impact

**Figure 6:**
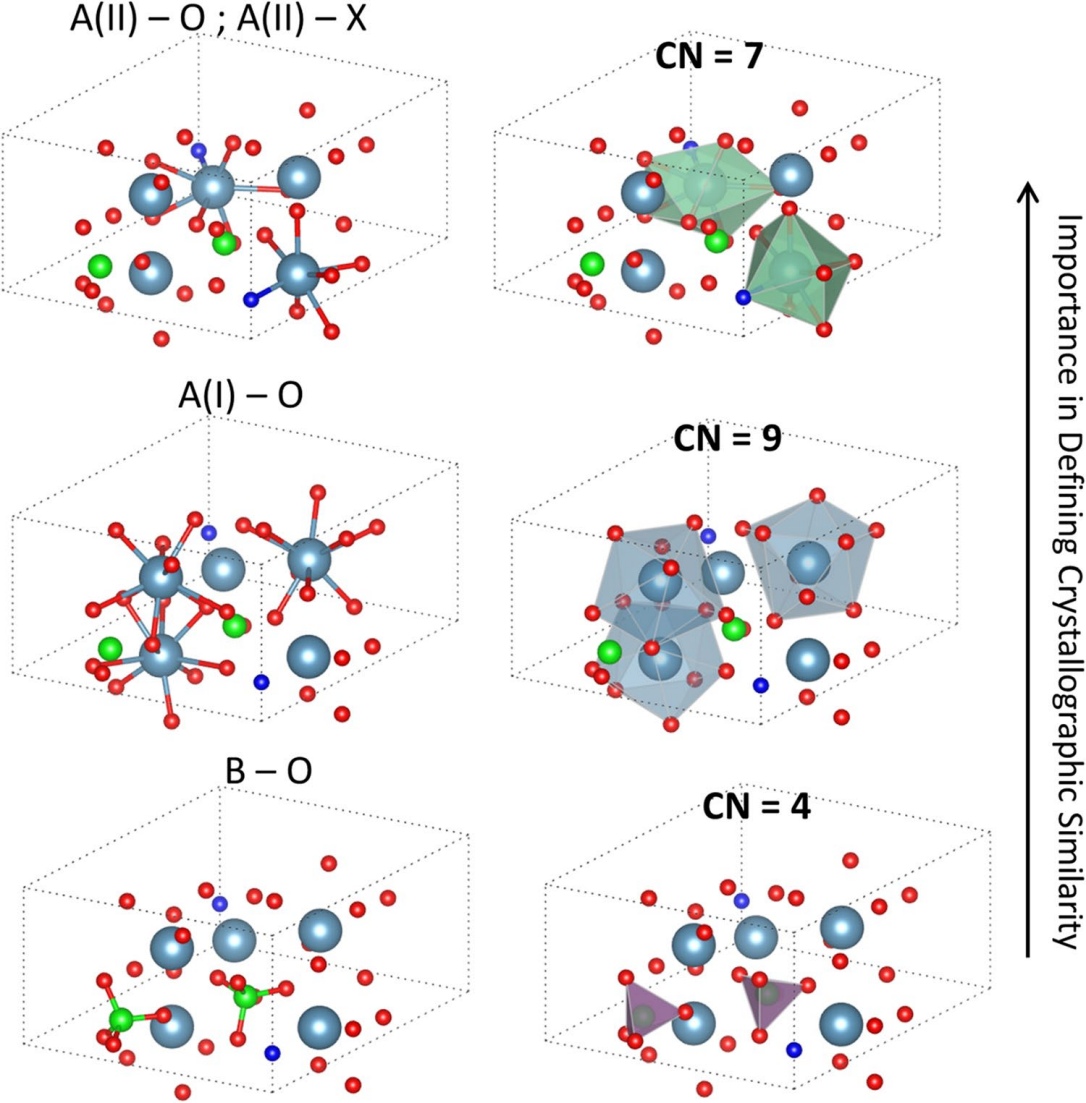
The coordination polyhedra constituting the apatite crystal structure. Through the use of TDA and tracking the changes as the barcode evolves, we have identified these relationships independent of the prior definition of structures. We find that the initial classification is based on the change in the X atom, i.e. impacting the coordination associated with A–X bond (CN = 7). Further connections then evolve which capture the other classifications (first A–O bond with CN = 9 and then B–O bond with CN = 4). The ordering of these classifications and chemical groupings allowed us to define the new genre of complex compound classification. The large blue atoms are A-site, the green atoms are B-site, the dark blue atoms are X-site, and the red atoms are oxygen.

From this table and the comparison of two approaches, we can make some critical interpretation of what is driving similarity. That is, by tracking the change in information, and the underlying causes, compared to a static representation provides a new series of insights on what constitutes the make-up of a crystal. The main factor captured in this analysis is the idea of the coordination polyhedra driving the similarity, as opposed to the PCA + k-means case which was governed by a pair of distortion angles. The three coordination polyhedra which dictate the TDA ordering are shown in Fig. 6. When compounds have common A and X elements (as is the case for Ca5(CrO4)3Cl and Ca5(VO4)3Cl, among others), that the A–X bond is the common characteristic. As the A(II) site is the site relevant for this bond, we can further include A(II)–O within this. Therefore, as shown, at this point in the barcode, the CN = 7 polyhedra shows up, and as it is the first to show up it is deemed the most important. This interpretation holds as when grouping on the fourth and fifth bars, the A-sites change but the X-sites are consistent. Thus, A–X bond is no longer a governing factor, and we can consider the other A–O bonds, namely corresponding with the A(I) site. Finally, we get classifications with same B-site chemistry, leading to the CN = 4 polyhedra which has B–O bonds as the edges.

**Figure 7:**
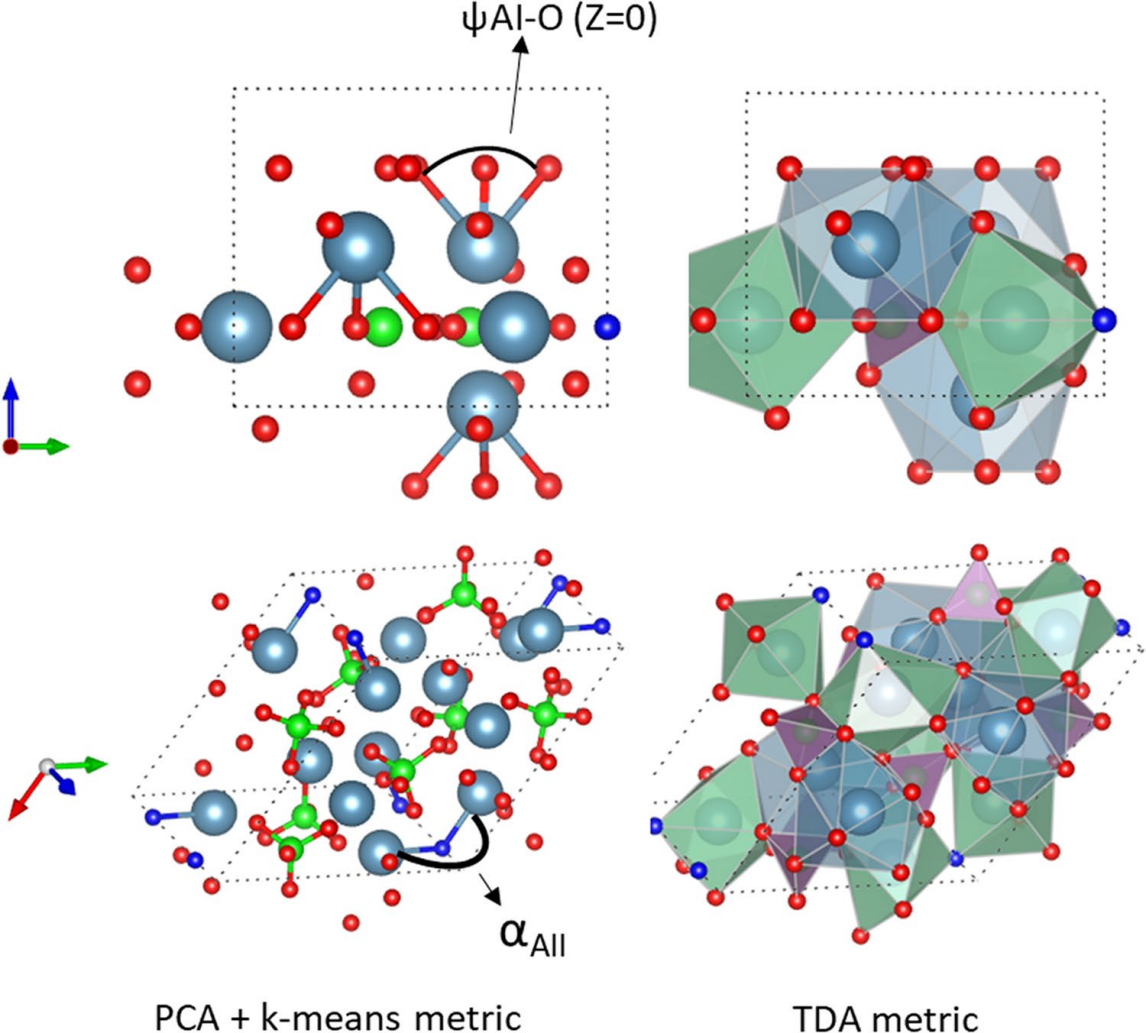
Comparison of the features identified as most critical from the prior PCA + k-means approach ^16^ and the TDA approach described here. In the case of the prior work, critical bonds are identified, while the TDA approach captures a more complete representation by accounting for the bonds and geometry of the coordination polyhedra. The bonds are seen to be represented in by the coordination polyhedra, with ψA1–O1 captured by the CN = 9 polyhedra (blue polyhedra), while α_AII_ shows up in the CN = 7 polyhedra (green). Neither bond shows up within the CN = 4 polyhedra (purple), indicating that the prior approach did not identify B site chemistry to have a major impact in defining similarity of chemistries.

From this, the importance of ranking is due most significantly by the CN = 7 polyhedra, which is made up of the A(II)–O and A(II)–X bonds. We determine this by finding the strongest criteria for similarity is compounds with common A(II)–O and A(II)–X bonds. This matches in some degree with the PCA analysis which included bonds related with the A(II) and X atoms, in addition to the A(I) atoms (Fig. 7). The second significant criteria is the CN = 9 polyhedra, which are comprised of A(I)–O bonds. Finally, the lowest ranking is for CN = 4 polyhedra which are comprised of B–O bonds. The twist angles used in the prior study did not take B–O bond angles into account, and so this is an added piece of information. Interestingly though the structure map selecting two angles involved a metric associated with the CN = 7 and CN = 9 polyhedra, and thus the two axes are related with the two most important aspects.

The TDA identified key bond pairs that were common between compounds and when we outlined those bond pairs, we found that the TDA was identifying the connections that outlined the basic structural building unit/polyhedra. Since the H_0_ topological class is tracking connectivity, it makes mathematical sense but also demonstrates that although we never input atom positions but did include chemical bonding information and bond geometry, the TDA in a totally unsupervised manner traced the connectivity of the atom positions that form the vertices of the coordination polyhedral. Also, the TDA analysis as visualized through the evolution of the barcode identifies the sequence of coordination number that drives clustering of compounds within the apatite family. This appears to be a means of comparing crystal structures, and capturing long-range structure within this particular family of apatites.

While the metaprism twist angle is a good indicator of classification for apatite structures ^22^, our earlier PCA work then showed that it is actually the pairing of twist angles that serve as the primary classification tool ^16^. This work here then shows that angular combinations provide one perspective of classification but the classification according to the role of certain CN polyhedral is the primary driving force for classification and that twist angle pairing serves as another level driving sub-classifications. The results described above using the TDA derived barcode representation of the persistence of correlations among descriptors has provided a rapid means of tracking classification based on simultaneously identifying the chemical environment around a given atom in a complex structure along with the overall arrangement of the neighbors around a given atom.

## Conclusion

The use of the TDA derived barcode representation of the persistence of correlations among descriptors has provided a rapid means of tracking classification based on simultaneously identifying the chemical environment around a given atom in a complex structure along with the overall arrangement of the neighbors around a given atom. The computational challenge in linking metrics that capture the geometrical characteristics of crystal structure and information on the chemical bonding that governs those spatial relationships revolves around the methods of partitioning spatial relationships in the three dimensions. This study has demonstrates the value of including TDA in the materials informatics toolkit and provided an example of its use in exploring and classifying coordination geometry by identifying the geometrical connectivity between atoms that form the structural building units has been discussed extensively in the literature. Many approaches have for instance used some form of Vornoi partitioning schemes to track coordination environments for a given atom ^34–36^. A key advantage of the approach described here is that it is robust enough to accommodate the different genres of crystal chemistry descriptors to identify the atomic environment and it permits one to automatically explore the persistence of classification schema among homologous compounds. Using the apatite family as a case study, we have been able to propose additional perspectives on classification of apatites by combining different approaches to data dimensionality reduction that harness both statistical learning and topological data analysis methods.

The present study serves to demonstrate the value of applying topological data analysis to explore the interactions of chemical and geometric descriptors of crystal chemistry to uncover new genres of classification rules for homologous compounds. We also show that combining different types of data dimensionality reduction techniques (eg. PCA and TDA) can offer new uses of machine learning methods as exploratory data mining tools. The TDA approach captured some of the same groupings along with new classification rules (associated with coordination number). Machine learning methods can in an unsupervised fashion discover coordination polyhedra geometry classifications in families of crystal structures. This is a topic of emerging interest in the materials informatics community, and we feel this work as contributing another approach to the field.

## Supplementary Information

Supplementary Information 1.

Supplementary Information 2.

Supplementary Video.

## Data Availability

The data and codes used in this study are available through the ‘Materials Data Engineering’ web portal (www.madeatub.buffalo.edu/MaDE@UB).
