# Supplementary material for: Classification of apatite structures via topological data analysis: a framework for a ‘Materials Barcode’ representation of structure maps
Source: Sci Rep. 2021 Jun 2;11:11599. doi: 10.1038/s41598-021-90070-4 (PMC8172868; doi:10.1038/s41598-021-90070-4)
Supplement: Supplementary file 1 — Supplementary Information 1. [file 41598_2021_90070_MOESM1_ESM.pdf]

Supplementary Section

Classification of apatite structures via topological data analysis: a framework for a 'Materials Barcode' representation of structure maps

Scott Broderick[1], Ruhil Dongol[1], Tianmu Zhang[1], and Krishna Rajan[1,*]

[1]Department of Materials Design and Innovation, University at Buffalo
*120 Bonner Hall, Buffalo, NY, 14260-5030, krajan3@buffalo.edu

1. Additional Information

- A file containing the complete barcode is provided. Each page of the file corresponds to the entire barcode at that particular filtration value.
- The following are figures to provide further clarity to the discussion in the main document. Figure S1 provides additional information in the connection between the input descriptor space and the bar code, while Figure S2 provides a more detailed structure map coming from the PCA analysis, used for comparison with TDA in the paper.
- In the main manuscript, the barcode representations were shown for H_0, which corresponds to the consideration of connected components within the input descriptor space. The reason for focus on H_0 and the more convenient interpretation were highlighted in the paper. However, we have included the barcode component resulting from H_1 in Figure S3. These classification results provide an assortment of patterns, when compared with the PCA and H_0 topological feature maps. For instance, the bulk of the groupings appear to be most sensitive to the mapping the phosphate compounds. However, other classifications include the detection of clusters of compounds that appear in the PCA analysis as part of a larger cluster of compounds.

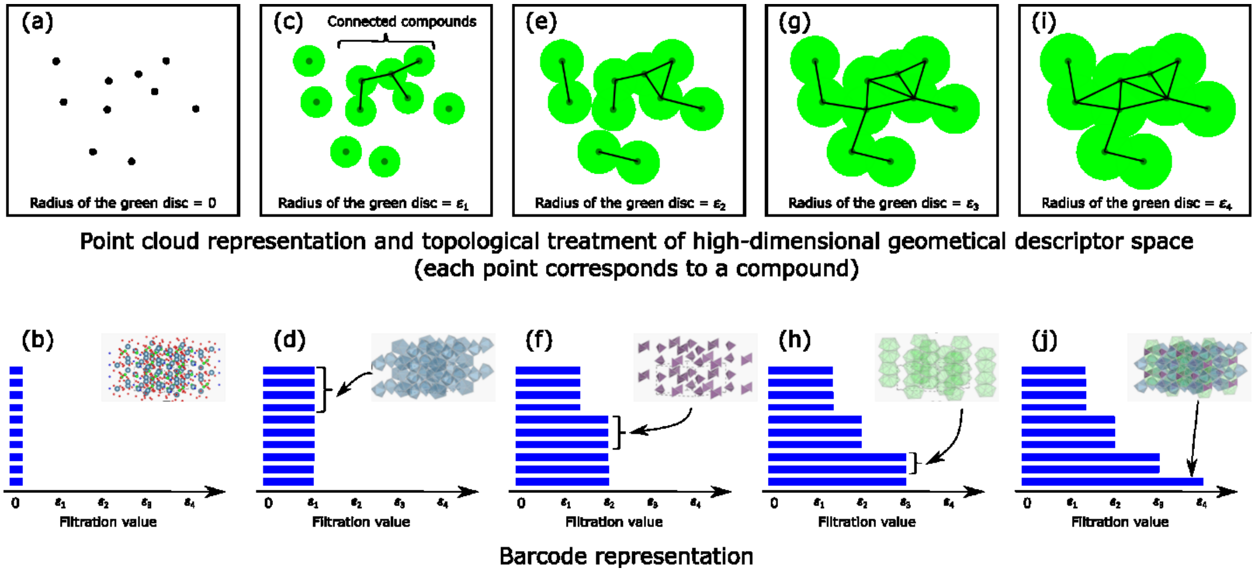

Figure S1. Graphical representation of the high-dimensional geometrical descriptor input space and the barcode representation. (a) Each point in the high-dimensional space represents an apatite compound. The birth of each connected components is 0 shown in the (b) barcode representation. The radius of the green disc increases to ε_1 (c), at which point five-compounds in the topological space are connected. (d) These connections correspond to the coordination associated with A-X bond (CN=7). Further connections then evolve which capture other classification, first (g)-(h) A-O bond with CN=9 and then (i)-(j) B-O bond with CN=4.

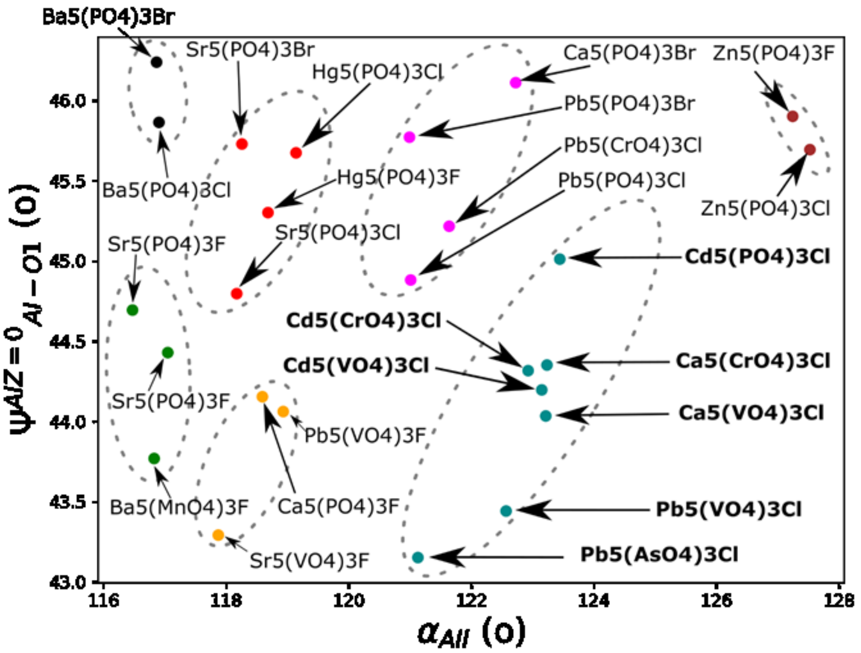

Figure S2. The structure map resulting from the PCA+k-means analysis. This includes all compounds labeled, while the version shown in the main document only labeled some of the compounds for clarity.

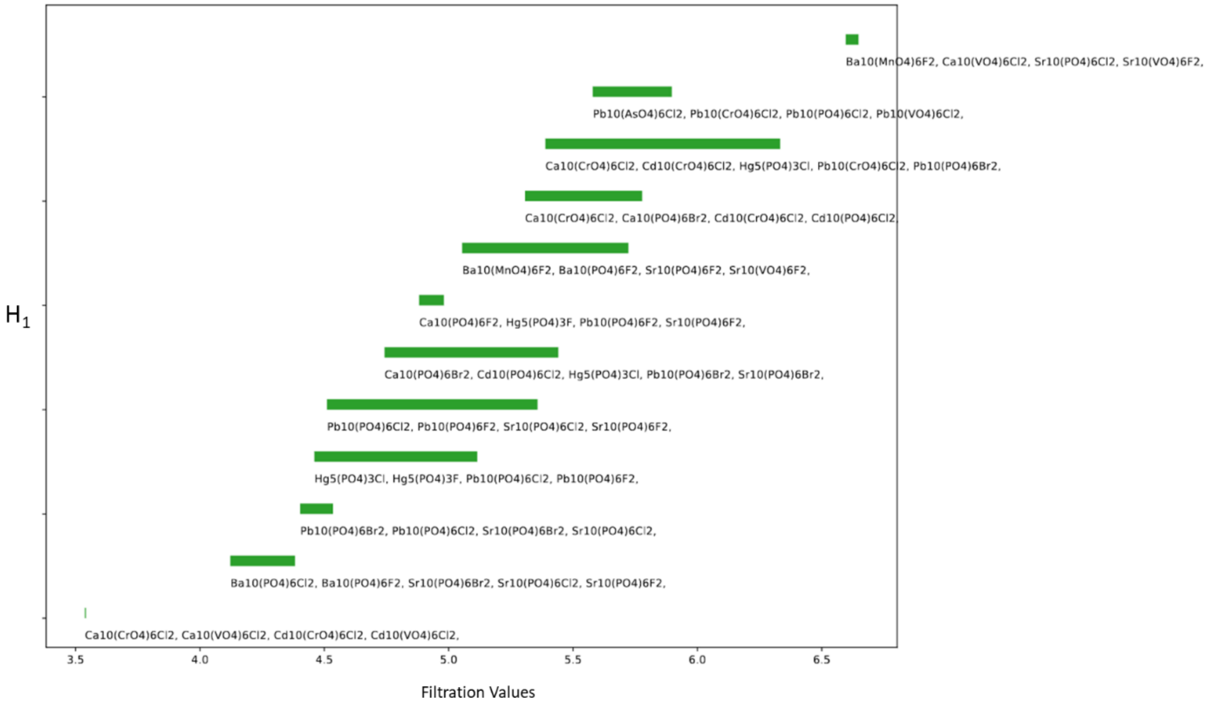

Figure S3. The final barcode corresponding to H_1. Note, this provides additional relationships not shown through H_0 as this instead is exploring tunnels within the descriptor space. This additional information is more difficult to link to the coordination and site-occupancy features, but also provides a different level of granularity in the analysis.

2. Persisitent Homology

In the following we further discuss the theoretical aspect of how to go from the input data to the final output. For the purpose of conciseness for the mathematical description below, the input apatites are denoted as [$Aptt_1$, $Aptt_2$, ..., $Aptt_{25}$] and we use $Aptt_{[i]}$ with *n* running from 1 to 25, with 10 descriptors used for this section and are denoted as [$Desp_1$, $Desp_2$, ..., $Desp_{10}$] and we use $Desp_{[i]}$ with *i* running from 1 to 10. Therefore one apatite in our dataset is represented as a 10-dimensional vector

$$Aptt_{[i,j]} = [Desp_{i,1},...,Desp_{i,j},...,Desp_{i,10}]. \quad (1)$$

In the Eq. 1, the index *i* is for 25 apatite and *j* is for 10 descriptors. To set up the frame of reference for the input data points, we treat each of the 10 descriptor $Desp_{[i]}$ as an independent dimension and standardize the values of that descriptor for all of the 25 input data points. This way the 25 data points can be treated as 25 points in some 10-dimensional Euclidean space IR^{10}.

a. Topological Data Analysis

Scale selection is ubiquitous in scientific research, it is commonly manifested as the determination of certain parameters which will have a effect on the results. Nevertheless, those value selections are commonly done arbitrarily or subjectively, this would result in the conclusion made being biased towards the selected value. As a general approach to be less biased, one could look at the set of results produced from a range of parameter values, and find the features that dominates. These features and the parameter values for them can be then used as a guide in the analysis afterwards. With point cloud data as the 25 points in IR^{10}, one can use homology to capture the topological features as elements in a group with the metric of the point cloud.

b. Simplices, Faces, Boundaries and Simplicial Complex

The first step in the persistent homology pipeline is to build a set of simplices from the input data points. In the following we ignore how to select a subset of the input points to form a simplex, which will become clear when filtration and persistence are discussed later in this section. Also the points are assumed to be in general positions. To define a simplex, we need a set of *n* points $\{v_1, v_2, ... v_n\}$ that are affinely independent in a Euclidean space, and a *n*-simplex can be then defined as the collection of the points *x* satisfying Eq. 3.

$$x = \sum_{i=1}^{n} t_i v_i, \quad \sum_{i=1}^{n} t_i = 1 \quad and \quad t_i \geq 0 \qquad (2)$$

Each of the v_i is a vector in Euclidean space of the form $[v_{i,1},...,v_{i,j},...]$ with *j* being the index for the dimensions of the space. As a simple example in R^2, three affinely independent points will define a triangle. In our case, the v_i's in Eq. 3 are the data pionts $Aptt_{[i]}$. Note in this case the index *i* does not necessarily cover the entire 25 numbers, it can be an arbitrary subset of integers from 1 to 25. Each of the $Aptt_{[i]}$ can be represented as 10-dimensional vector with the elements of the vector being the $[Desp_{i,1},...,Desp_{i,j},...,Desp_{i,10}]$, and the above equation becomes

$$x = \sum_{i=1}^{n} t_i Aptt'_i, \quad \sum_{i=1}^{n} t_i = 1 \quad and \quad t_i \geq 0 \qquad (3)$$

To maintain the generality of this introduction, we keep using the notation of v_i for the input data points.

A face of a simplex is the simplex spanned by a subset of the set of points that are spanning the original simplex. The boundary of a simplex is the collection of points for which at least one of the barycentric coordinates in Eq. 3 equals 0. It follows from the definition of the face of a simplex, the boundary is the union of all its faces with co-dimension of 1. An important property about boundary is that the boundary of a boundary is an empty set. For a 0-simplex (the trivial case), it does not have a boundary. For a 1-simplex, the boundary is the two end points which span it, and the boundary of the two end points is the same as the the union of the boundaries of the two points which is an empty set. The boundary of the 2-simplex is the union of three 1-simplices, and for each of the 1-simplex its boundary is two 0-simplices, so we are left with 3 0-simplices with each of multiplicity 2. Under Z/2Z arithmetic the multiplicity of 2 cancels the the node, and left with an empty set. Throughout this paper we will use the Z/2Z arithmetic, general case will require the introduction of the orientation of the simplices which can be found in text regarding simplicial homology.

A Simplical Complex is a collection simplices such that the intersection of two simplices is either a face of both of the simplices or an empty set. Geometrically this restricts how simplices can be joined together to form a simplicial complex in Euclidean space IR^n.

i. Chain Groups and Homology Groups

Simplices can have positive or negative signs, we can further extend them algebraically to allow them to have multiplicities, i.e. to assign an integer coefficient to every simplices. In a simplicial complex, a *k*-chain is a collection of *k*-simplices with +/- sign indicating the orientation and an integer indicating the multiplicity. All of the *k*-chains with the operation of formal sum is a group, called chain group and is denoted as $C_k(K)$. It can be shown that this group is a free Abelian group. In the following we will define the boundary operator which when applied to a chain will give us the boundary of the chain.

First note that for an oriented simplex $\sigma = [v_1,...,v_p]$ with $p > 0$, we can obtain one of its faces of co-dimension 1 by removing one of the vertices from the vertex set expression. To keep track of which vertex is being removed, instead of deleting it from the vertex list, it is denoted with a ˆ sign in the list. For example the simplex $\sigma = [v_1,...,v_p]$ with the vertex v_j removed is written as $\sigma = [v_1,...,\hat{v}_j,...,v_p]$. This is also because we need the positional information of the removed vertex to determine the orientation of the face. The orientation of the obtained face should conform with the original simplex, for this a parity term is added to the expression of the face. The face is then expressed as $\sigma = (-1)^{j-1}[v_1,...,\hat{v}_j,...,v_p]$.[1] Following this, the boundary operator is defined as:

$$\partial[v_1,...,v_p] = \sum_{i=0}^{k}(-1)^{j-1}[v_1,...,\hat{v}_j,...,v_p] \qquad (4)$$

The chains with no boundary are called cycles. For all of the elements in the group $C_k(K)$, there are two types of cycles: the ones that are surrounding a vacant region and the ones that are not. To find the topological features in the simplicial complex, it suffice to find the cycles/chains that are enclosing the vacant regions. We call the cycles that are differed only by simplices homologous to each other, and the cycles that are homologous to each other fall into the same class. Therefore to identify the topological features we need to identify the classes of cycles, and these classes are the elements of the homology group.

Recall that in general the result of the boundary operation on a *k*-chain is a $(k-1)$-chain or empty set. This means the boundary operator introduces a homomorphism from the k chain group to the $k-1$ chain group. So we add subscript to the operator when necessary to denote the dimension of the chain group the operator is operating on, i.e., $\partial_k : C_k(K) \to C_{k-1}(K)$. Thus, the cycles in the *k*-chain group is the kernel of the homomorphism introduced by the boundary operator ∂_k, denoted as $Z_k(K)$. And, for the chain group of dimension k, all of the elements except the identity are in the image of the boundary operator ∂_{k+1} which is denoted as $B_k(K)$. It can be shown that $Z_k(K)$ is a subgroup of the *k*-chain group and $B_k(K)$ is a subgroup of $Z_k(K)$. With these two subgroups defined, we apply the quotient operation of the subgroups to identify the topological features. In particular, the subgroup $B_k(K)$ contains the elements for which if two cycles are differed from, they will be in the same class. This is the subgroup used to set the equivalence while $Z_k(K)$ contains all of the cycles, and the definition for the homology group $H_k(K)$ is:

$$H_k(K) = Z_k(K)/B_k(K). \qquad (5)$$

ii. Vietoris-Rips Complex, Persistence Module and Barcode

The topology of the set of discrete points is trivial. Vietoris-Rips complex is a multi-scale combinatorial object which by applying homology to will provide us with the topological features of the point cloud at

different scale. To construct a Vietoris-Rips complex from a set of points in R^N, we associate each of the points to a N-disk and continuously increase the disk radius from 0. The set of points are the 0-simplices of the complex. As the disk radius increases, they will start to have non-empty intersections. For k points, when their disks have mutually non-empty intersections, we add a k-simplex which is spanned by the k points to the complex. It is worth noting that events like this will keep happening at certain values of the disk radius. This creates a series of simplicial complexes, and each of them is a subcomplex of its succeeding one.

$$\emptyset \subset K_0 \subset K_1 \ldots \subset K_n = K \quad (6)$$

This further also a map from each simplicial complex to its successor by the inclusion map, therefore this is also a filtration.

$$\emptyset , \rightarrow- K_0 , \rightarrow- K_1 \ldots , \rightarrow- K_n \quad (7)$$

The idea behind the sweeping of radius from 0 is to create a series of simplicial complexes, such that the topological features can be examined in the filtration, reduce the biases towards selecting different radius values.

Applying the homology to the complexes in the filtration gives us a series of homology groups (for every dimension). This series of homology group is also connected by homomorphisms introduced by the inclusions of the complexes. The elements of the homology groups are classes of homological features (i.e., cycles), assuming the simplicial complexes have finite number of dimension k topological features, then the group will have a finite set of basis elements (classes). For the different groups in the filtration, they may have different basis classes (elements) and, for a class it can start to appear in the i-th group and either end its appearance at the j-th (with $i < j$) group or continue to present to the last group. This defines the “birth” and “death” and thus “persistence” of a basis element in the sequence, and we have the definition for the persistent homology group:

$$H_k^{i,p} = Z_k^i / (B_k^{i+p} \cap Z_k^i). \quad (8)$$

In this equation, the k indicates the homology dimension, i and p indicate the indices of simplicial complexes in the filtration. This is called the *p-persistent homology group of* K^i, and the maps from one homology group to the next is just the special case with $p = 1$. To track the persistence of a basis element, we need to identify the maps from one group to the next. Recall the definition of the k-chain group which is a free abelian group with the integer Z. To compute the persistent homology, we view it as a free Z-module and this allows us to change the ground rings. Some authors use the notation $H_k(K,\mathrm{Q})$ for the homology groups where Q is used to indicate the ground ring. In particular, we will use a graded polynomial ring for the computation. A Persistent Module is defined as $M = \{M^i,\phi^i\}_{i\geq 0}$ over the ring R, where the M^i’s are the R-modules and the maps ϕ^i are the homomorphism from M^i to M^{i+1}. Figure 1 illustrates the idea. In this figure, every node M is a module where the superscript denotes its index of the simplicial complex in the filtration and the subscript denotes the dimension of the homology. The boundary operators maps the modules in the vertical direction and in the horizontal direction the homomorphisms map the modules introduced by the filtration.

There is a categorical equivalence between the categories of the persistent module of finite type over R and the finitely generated non-negatively graded module over $R[t]$. Specifically, when the ring is $R[t]$ with standard grading, the persistent module can be written as $\bigoplus_{i=0}^{\infty} M^i$ together with the action of t for shifting the grade upwards:

$$t \cdot (\alpha^0,\alpha^1,\alpha^2,\ldots) = (0,\phi(\alpha^0),\phi(\alpha^1),\phi(\alpha^2),\ldots). \quad (9)$$

By this we have combined a series of modules into one graded module. When the ground ring is a field F, we can apply the structure theorem for finitely generated modules over a principal ideal domain to obtain the decomposition of this algebraic structure:

$$\left(\bigoplus_{i=1}^{n} \Sigma^{\alpha_i} F[t]\right) \oplus \left(\bigoplus_{j=1}^{m} \Sigma^{\beta_j} F[t]/(t^{\gamma_j})\right) \quad . \qquad (10)$$

$F[t]$ is the graded ring over the field F, the Σ^i denotes the the upward shift of the grading by i, and the (t^j) is the ideal generated by t^j. Intuitively, when the upward shift Σ^i is applied to the ring $F[t]$, the ring will have no elements contain the terms of $F, Ft, Ft^2, \ldots, Ft^{i-1}$. Similarly, the ideal (t^{γ_j}) will have no elements contain the term of grade lower than $j-1$. So a component in the free part of the decomposition in Eq. 2.3.2 represent a basis element starting from i and persist through the filtration; a component of the torsion part represent a basis element which starts at i and ends at j in the filtration. This information can be convenient represent by a set of horizontal bars on the scale of grading. A component of the free part corresponds to a bar starting at the amount of its upward shift grade and does not end. A torsion component corresponds to a bar starting at the amount of upward grade shift and ends at the grade of the PID.
