# Supplementary figures and images for: Classification of apatite structures via topological data analysis: a framework for a ‘Materials Barcode’ representation of structure maps
Source: Sci Rep. 2021 Jun 2;11:11599. doi: 10.1038/s41598-021-90070-4 (PMC8172868; doi:10.1038/s41598-021-90070-4)

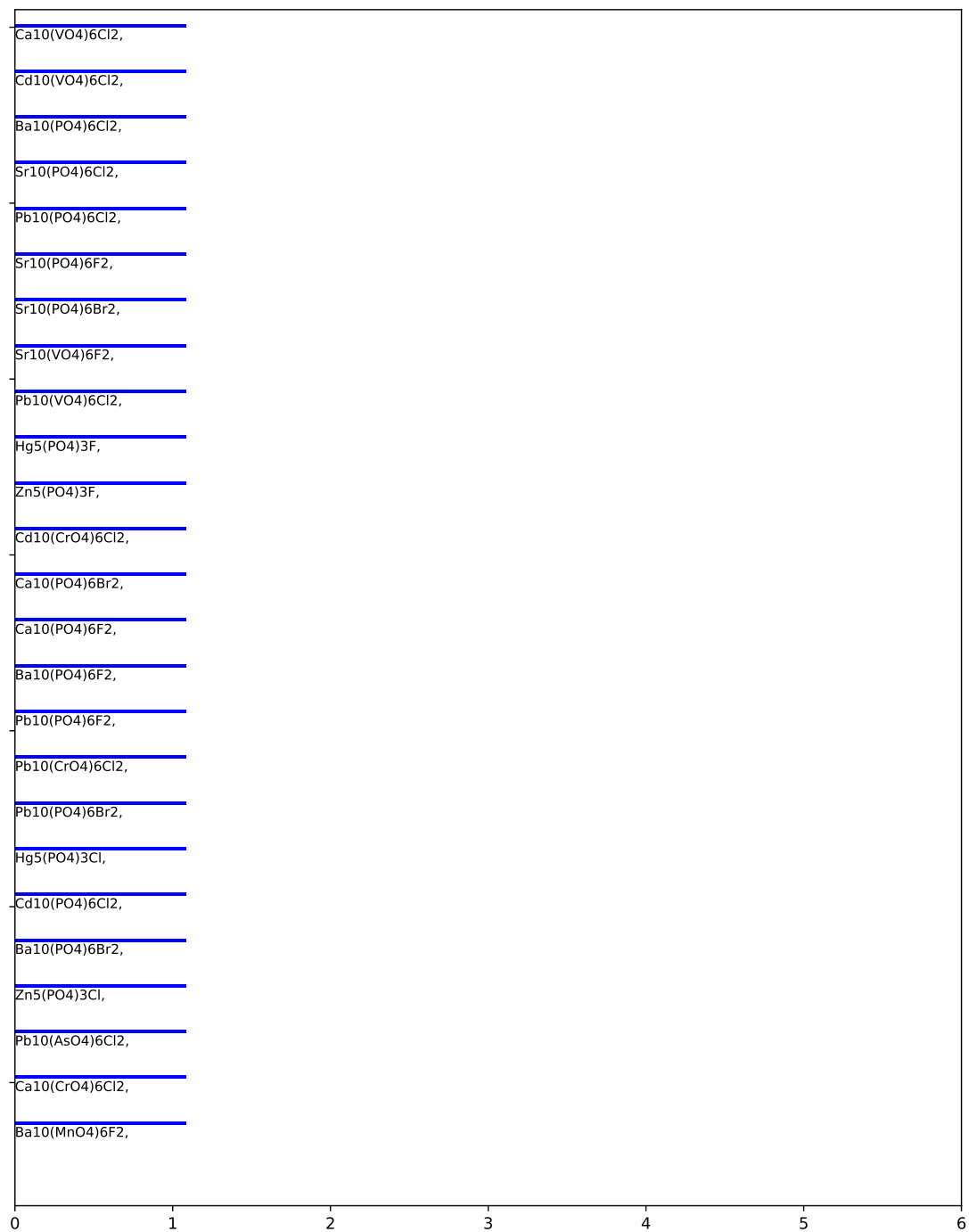

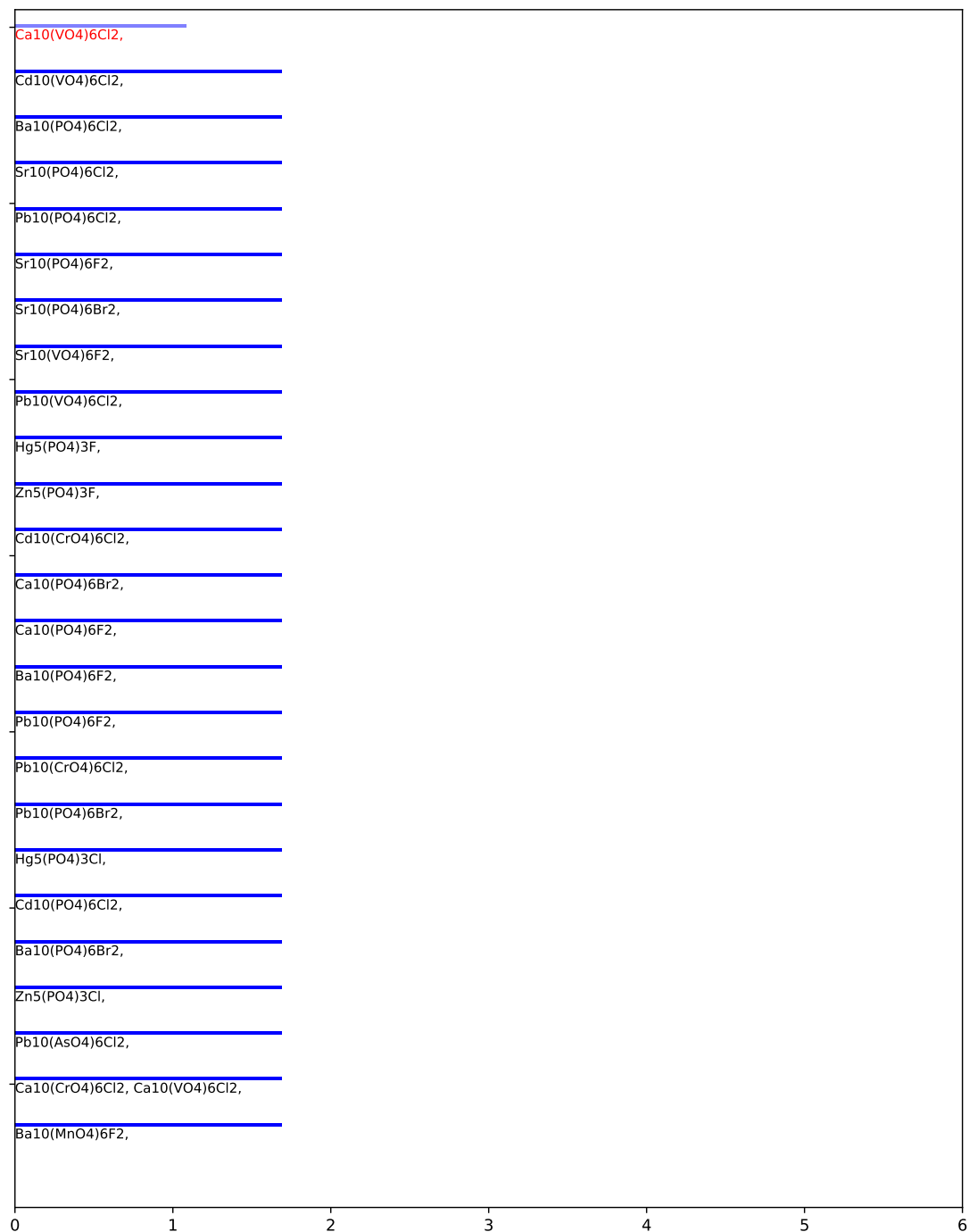

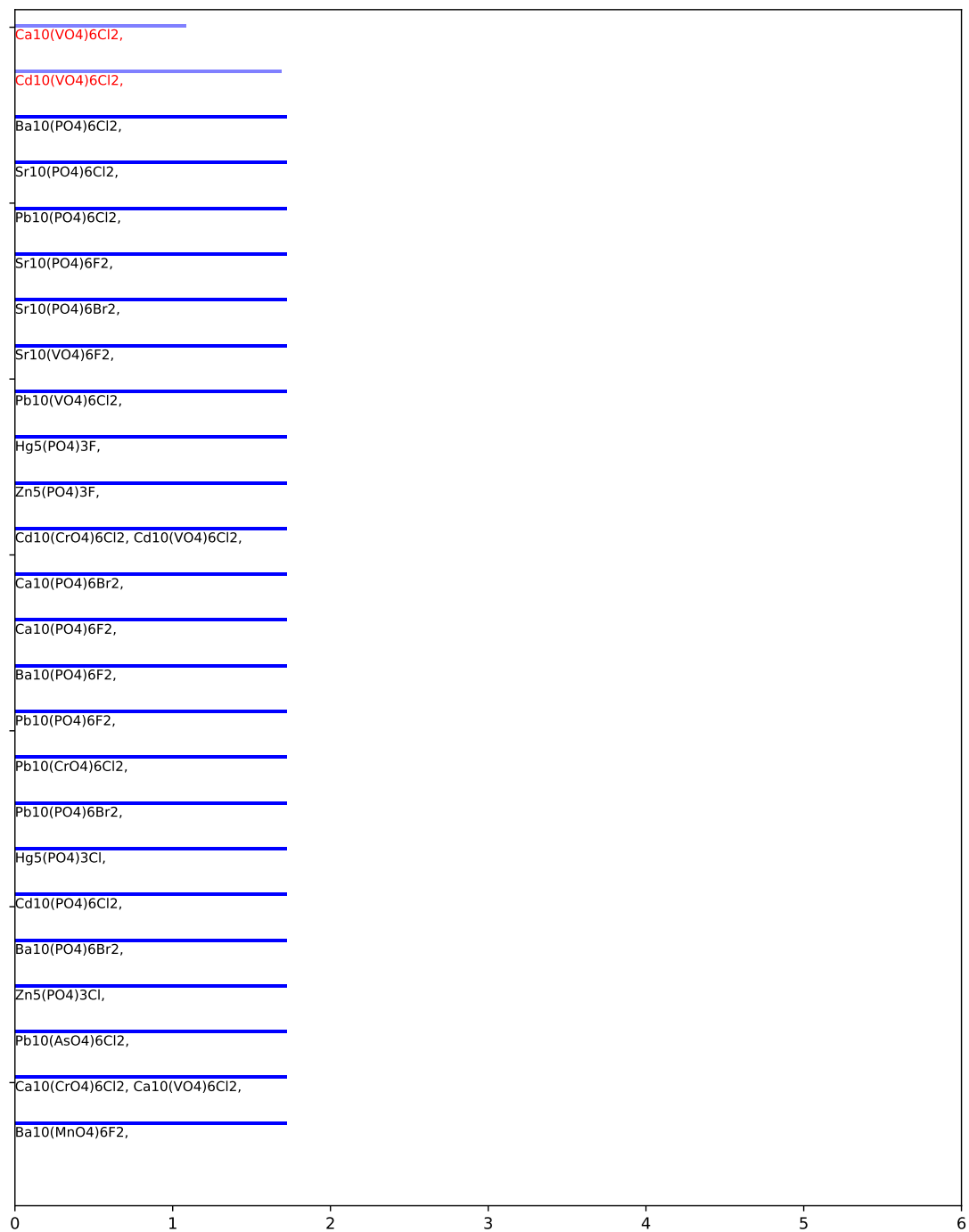

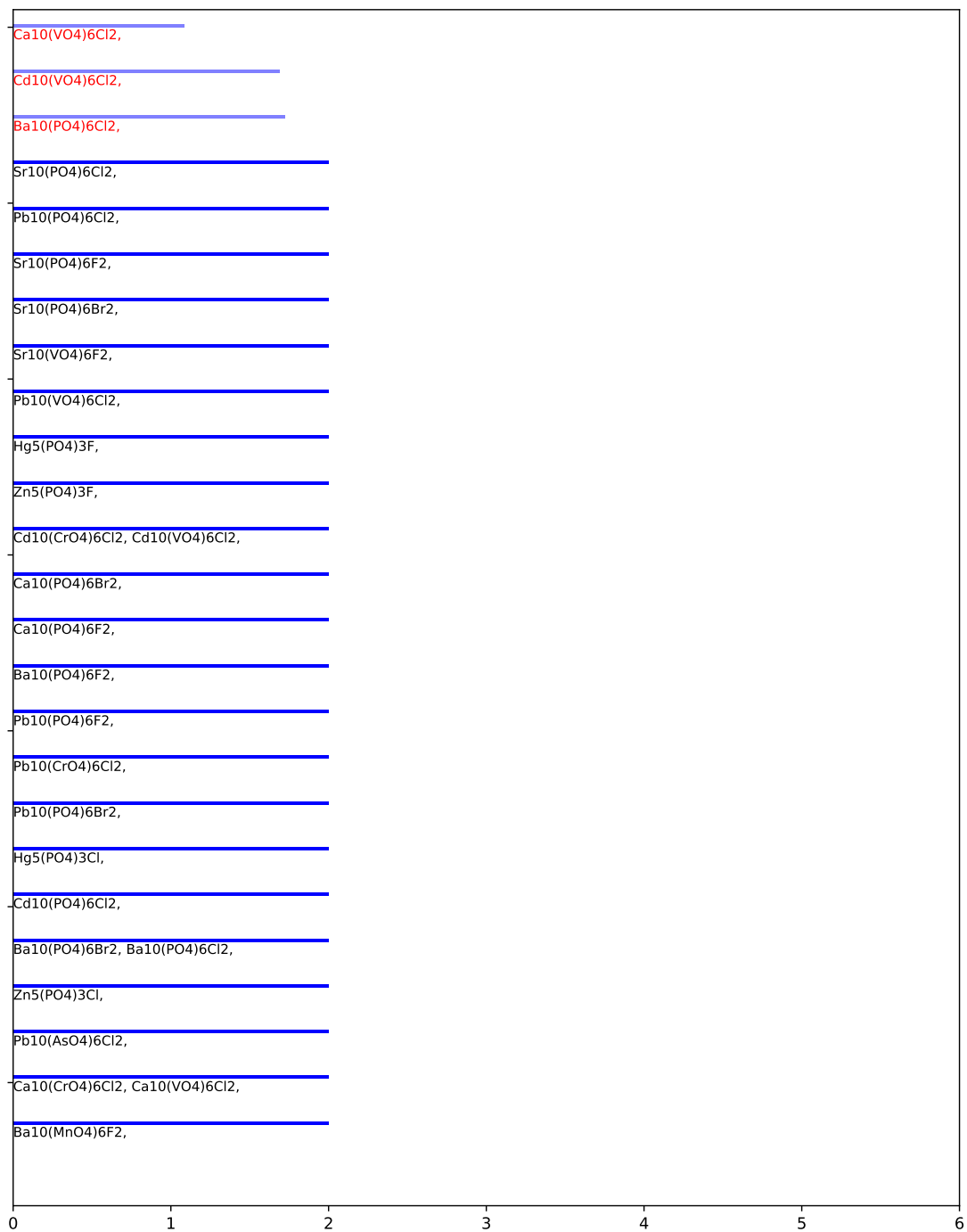

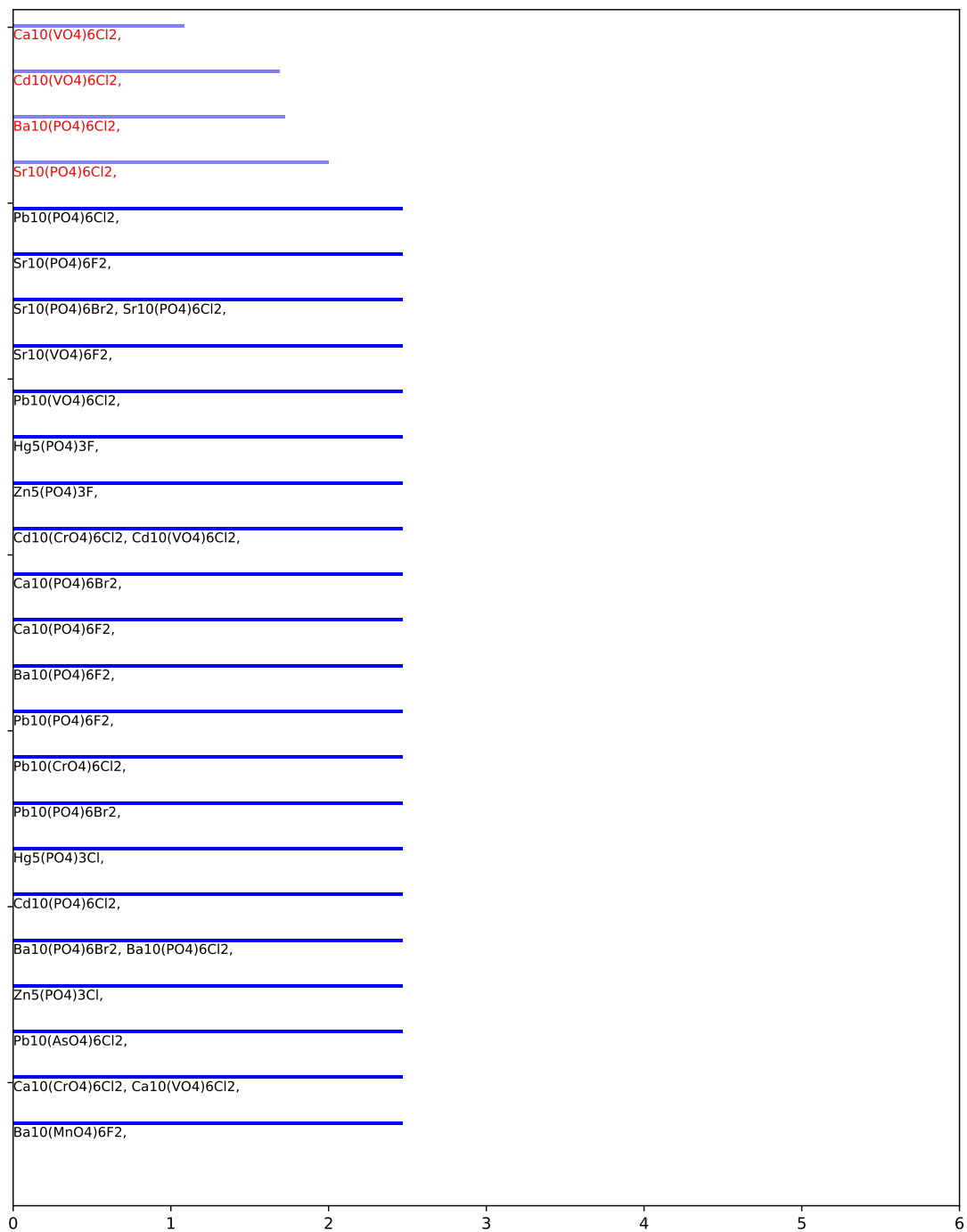

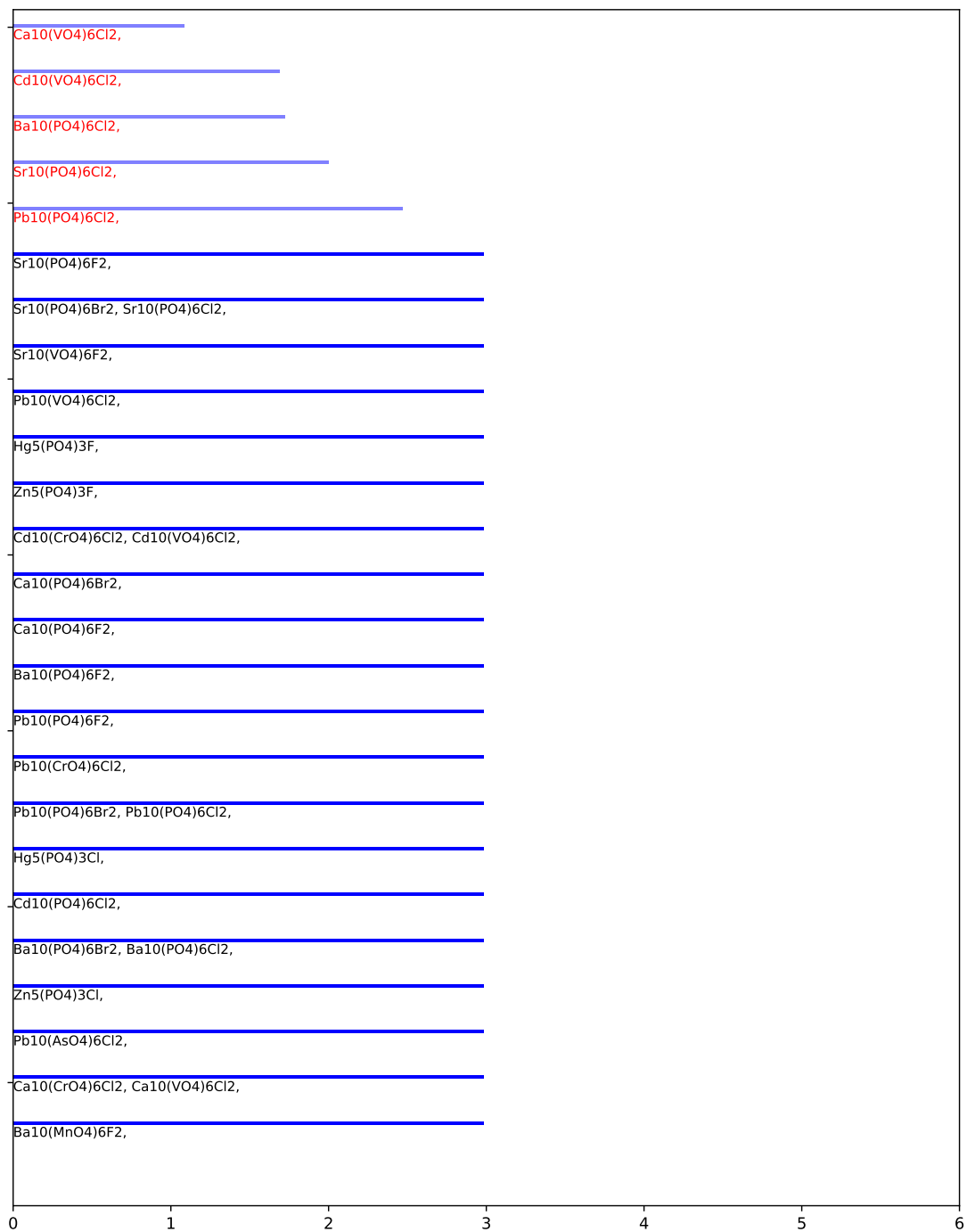

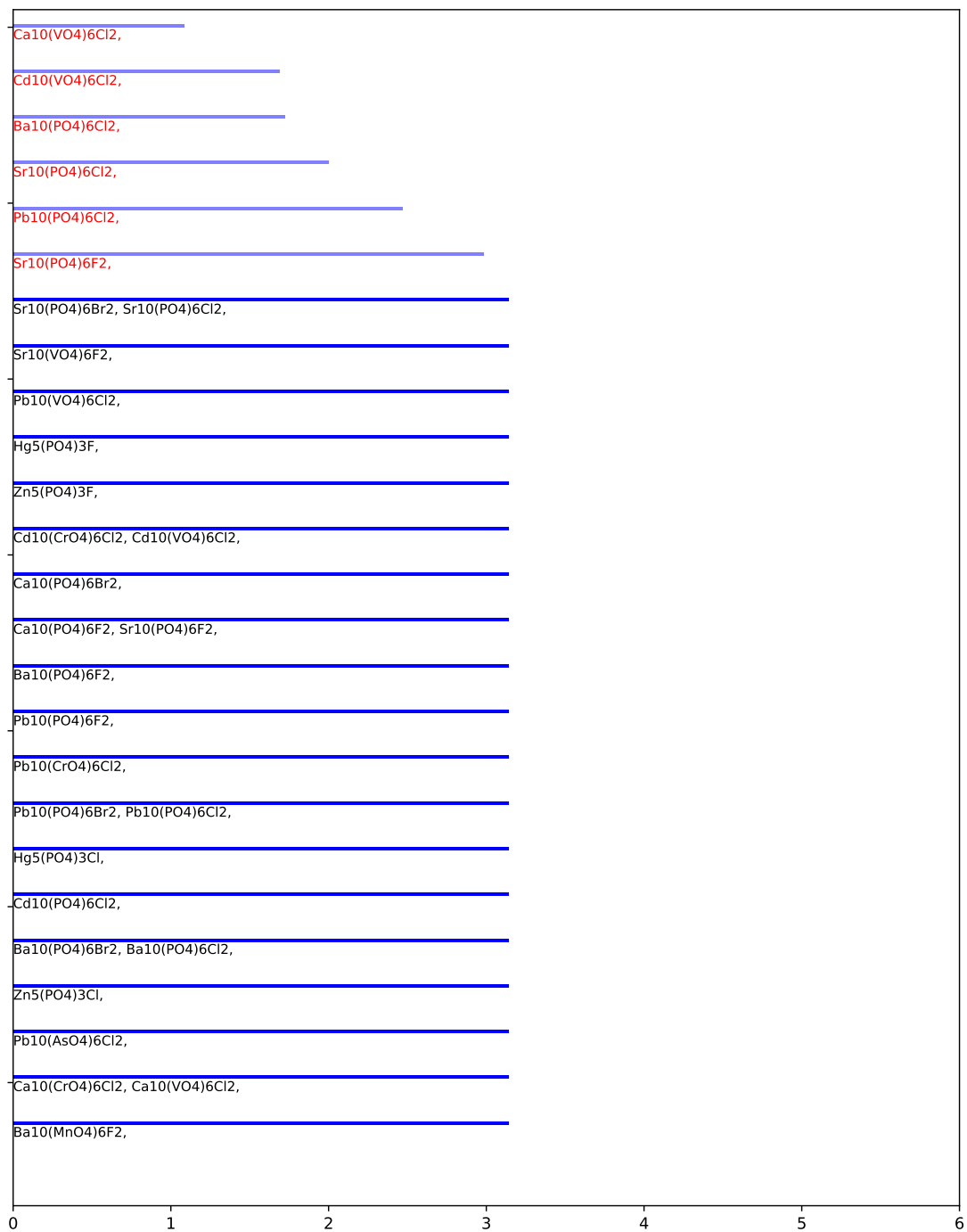

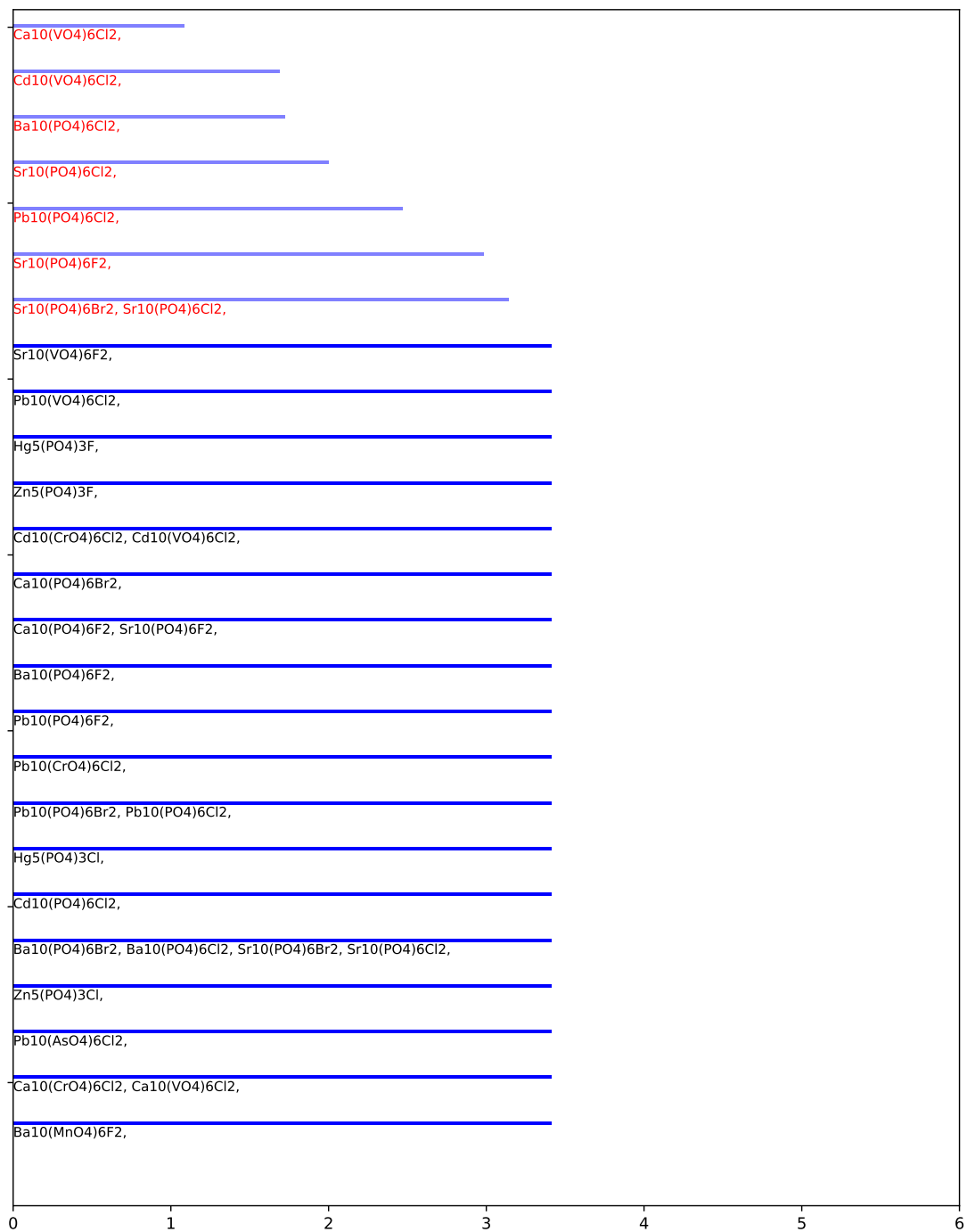

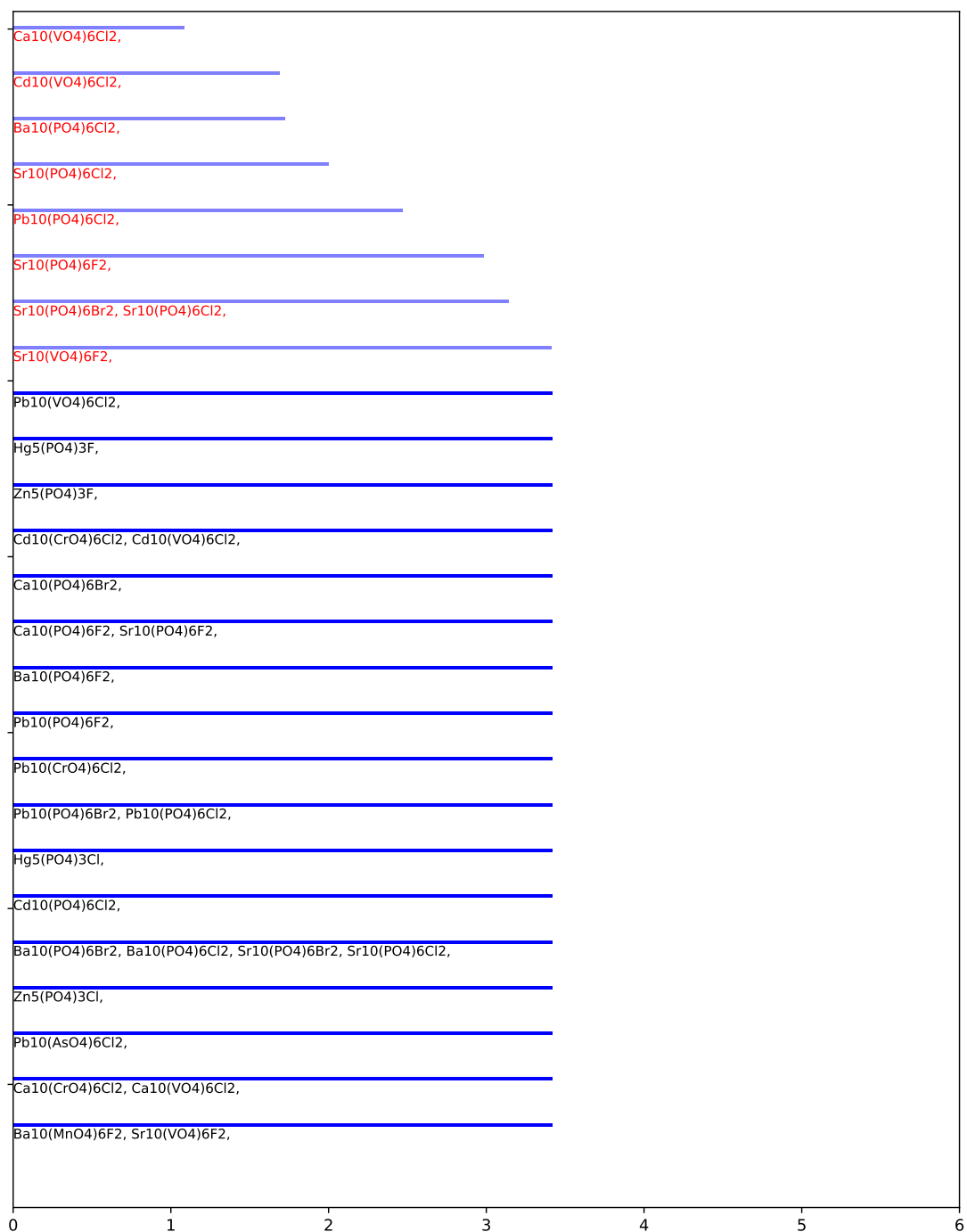

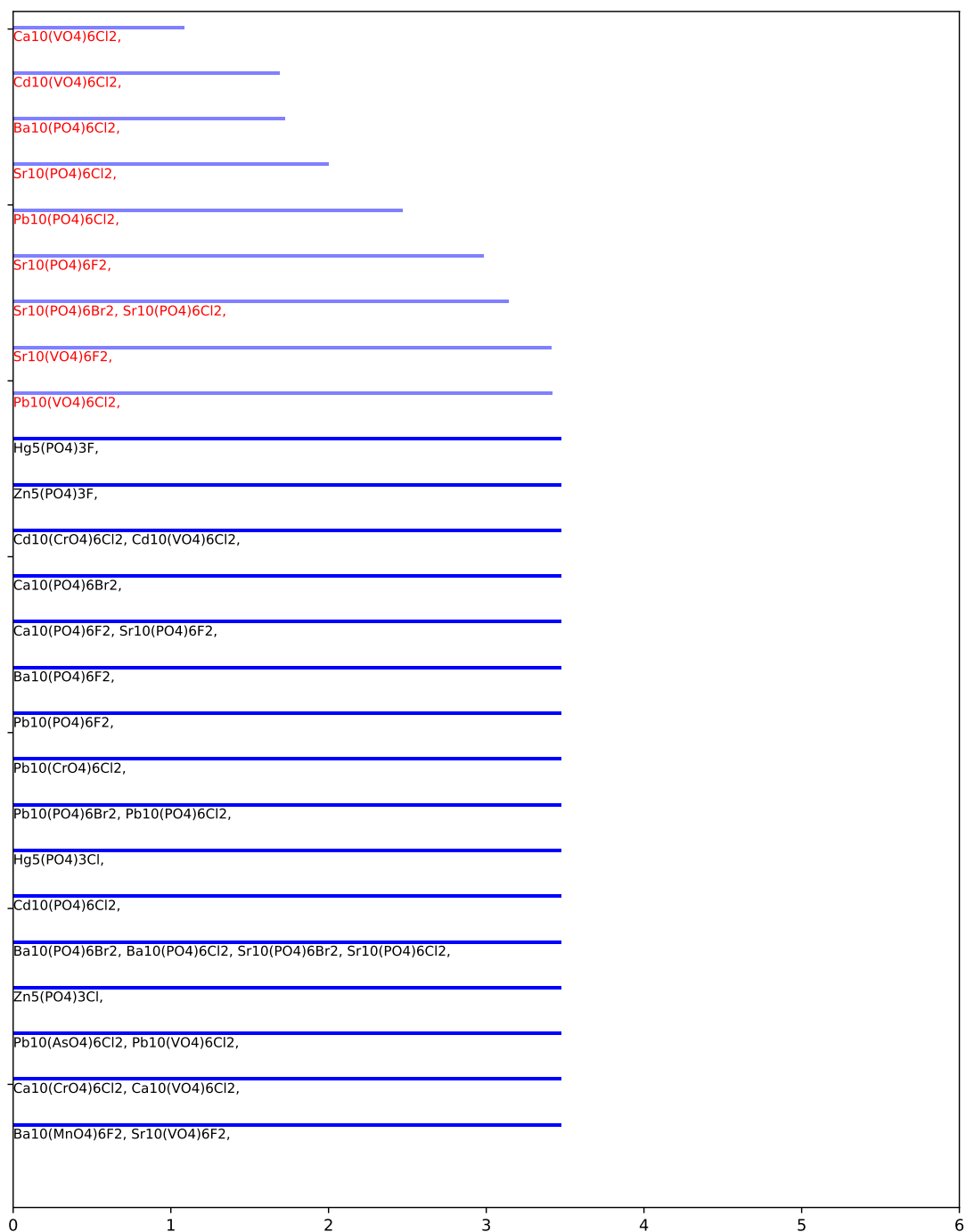

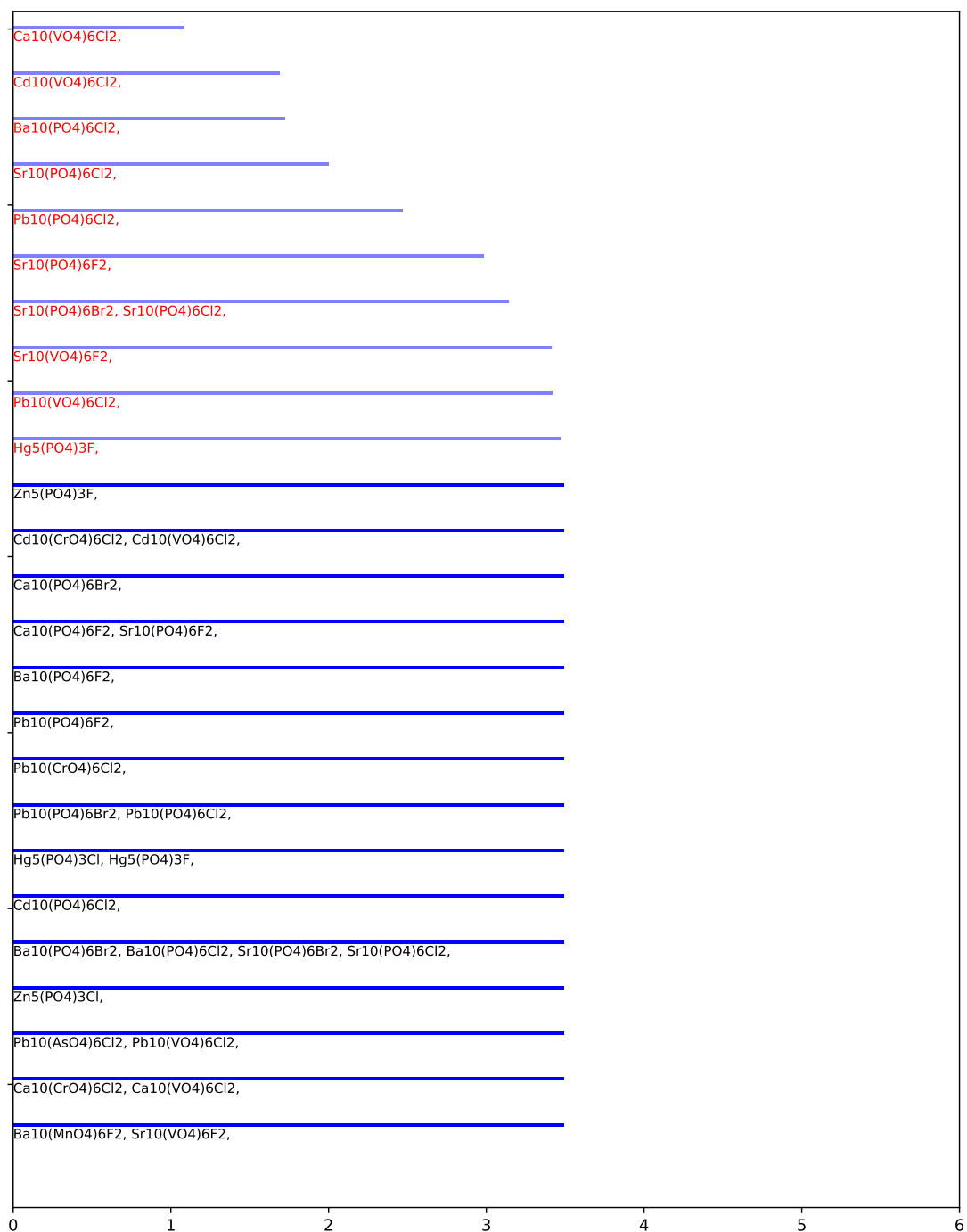

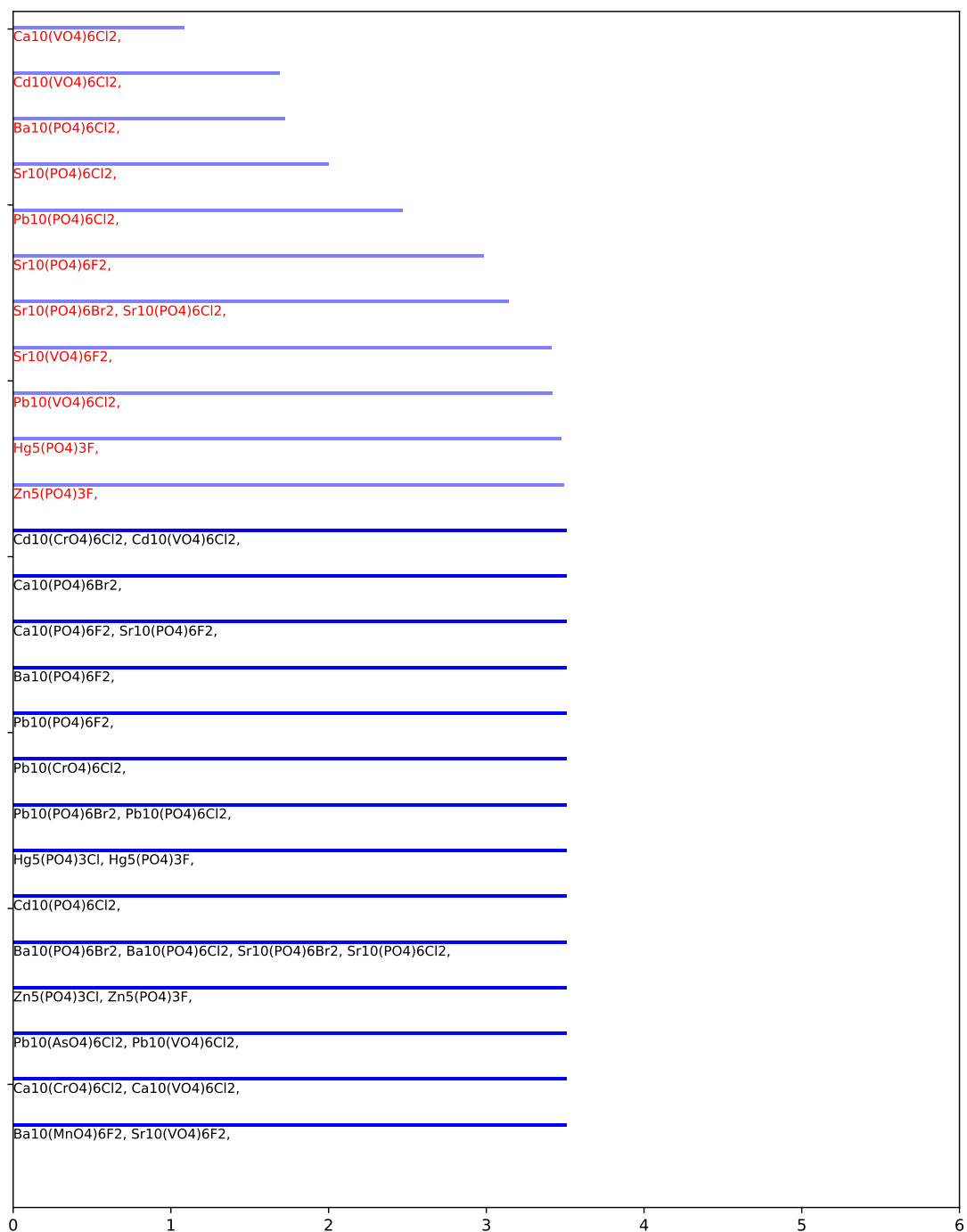

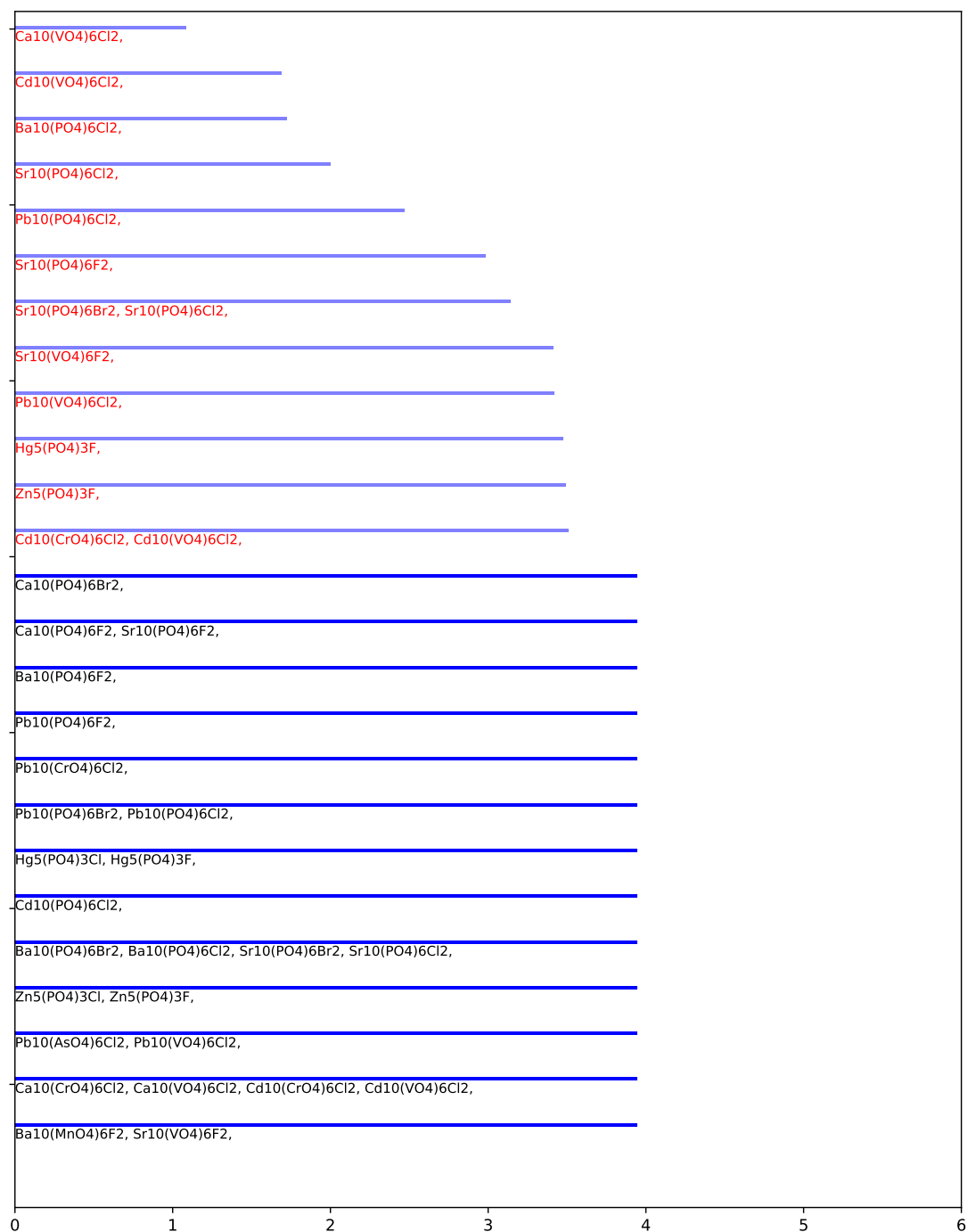

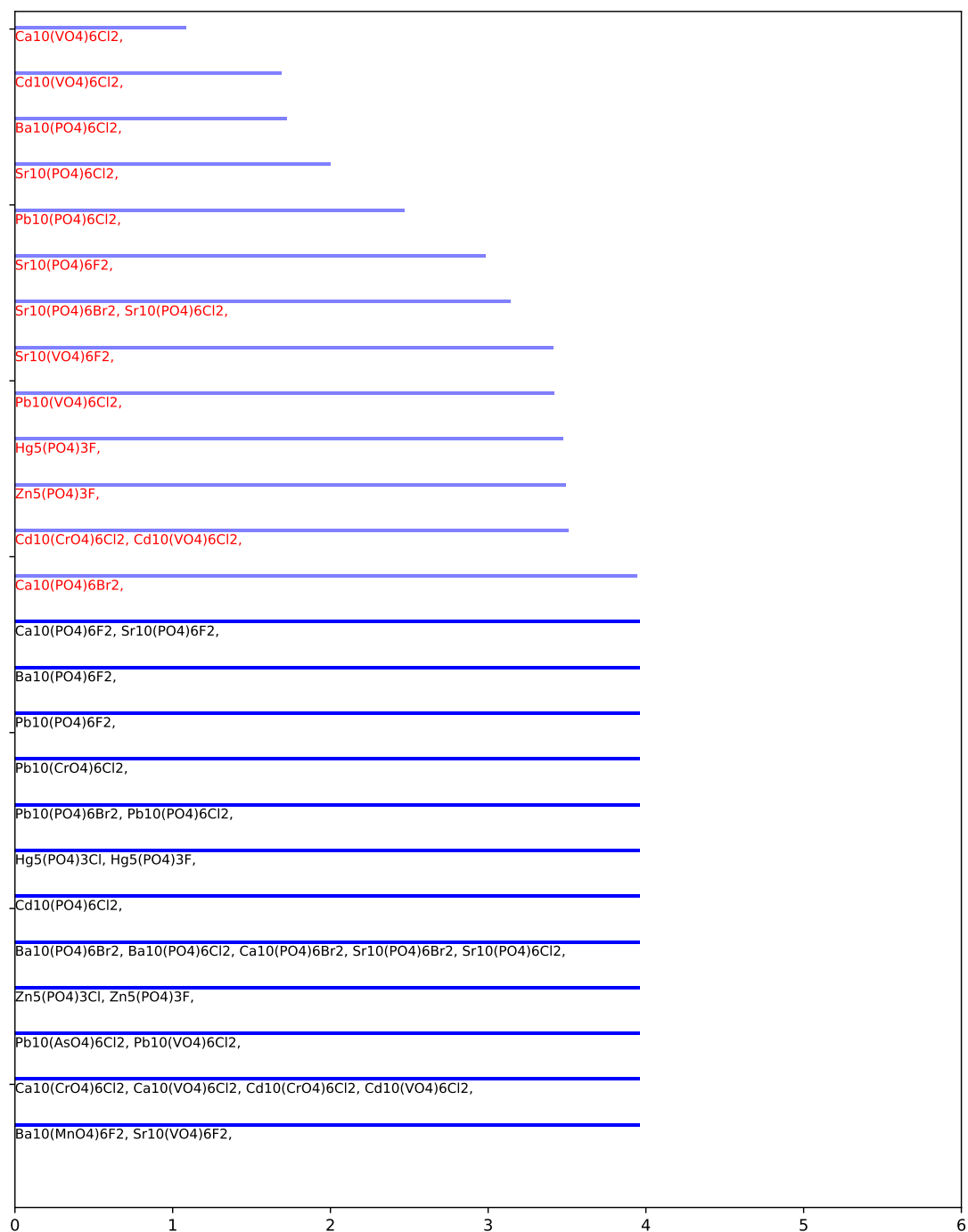

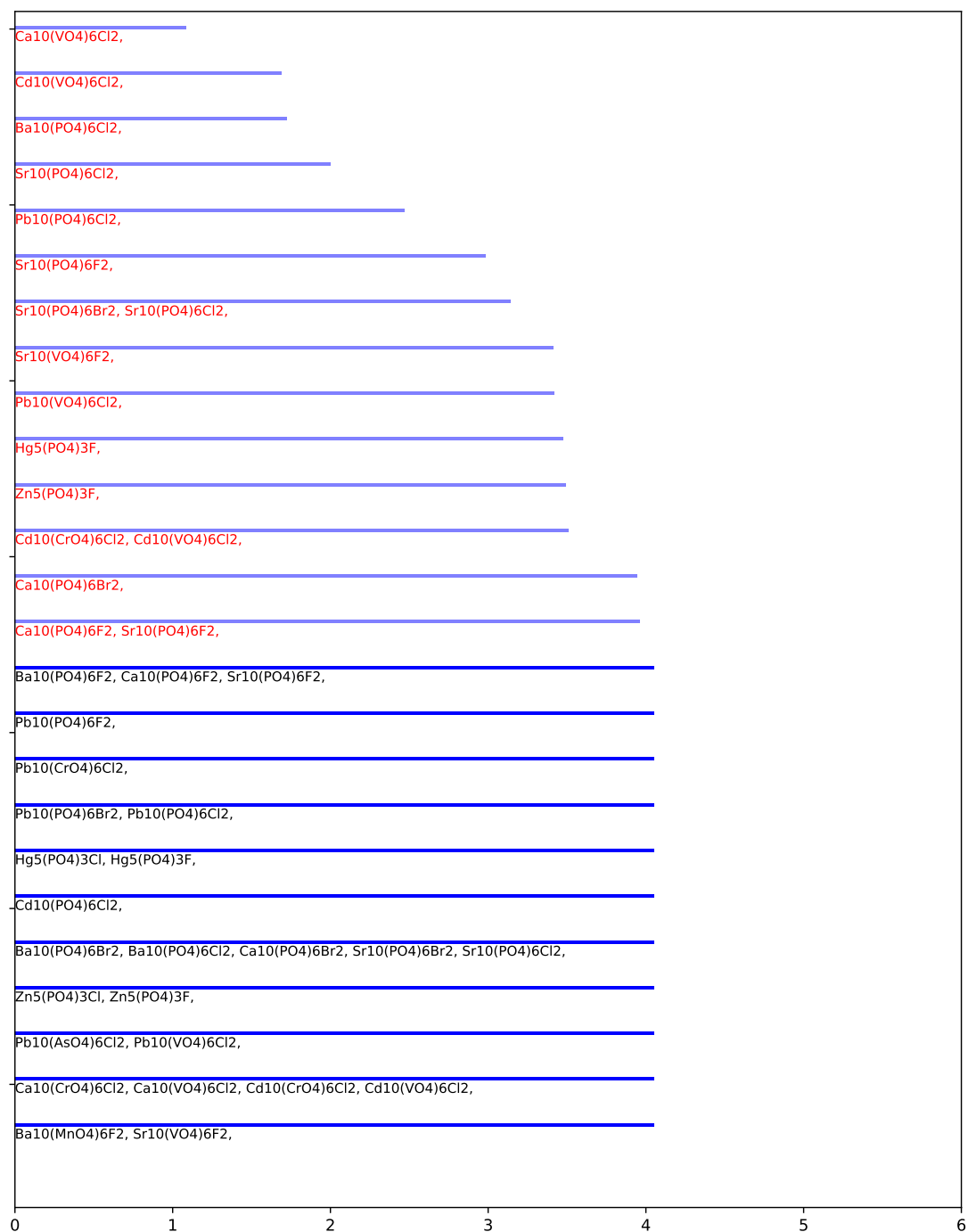

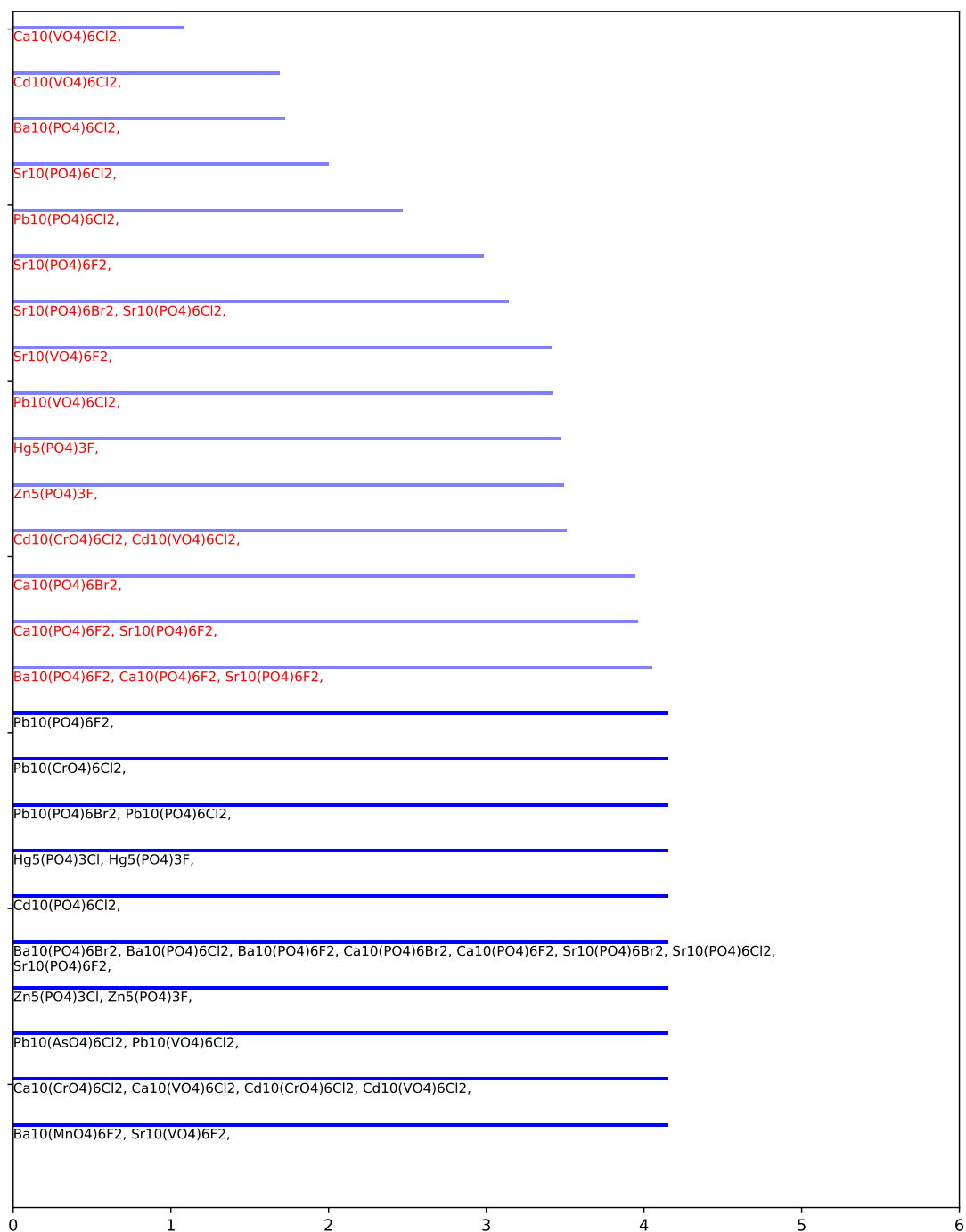

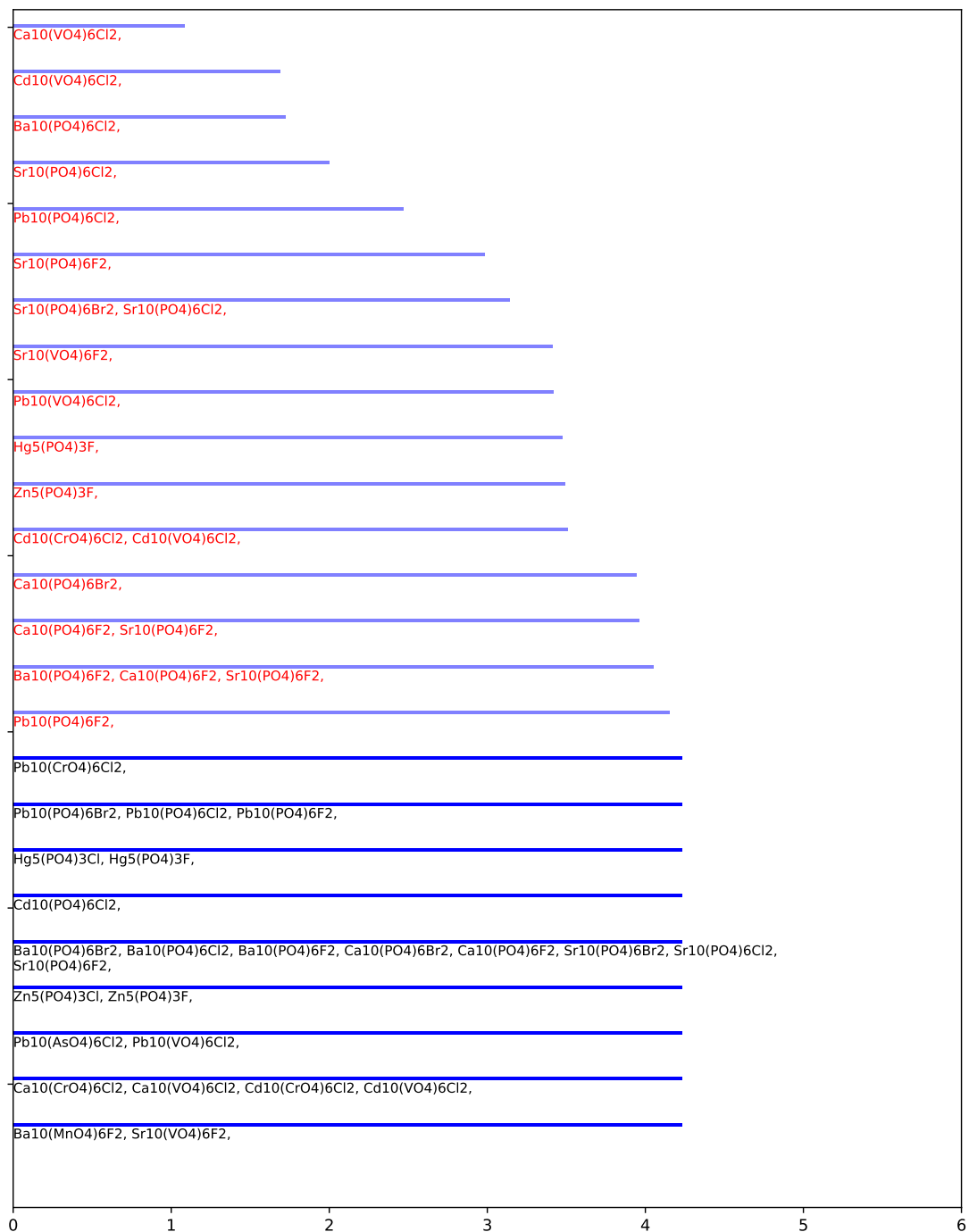

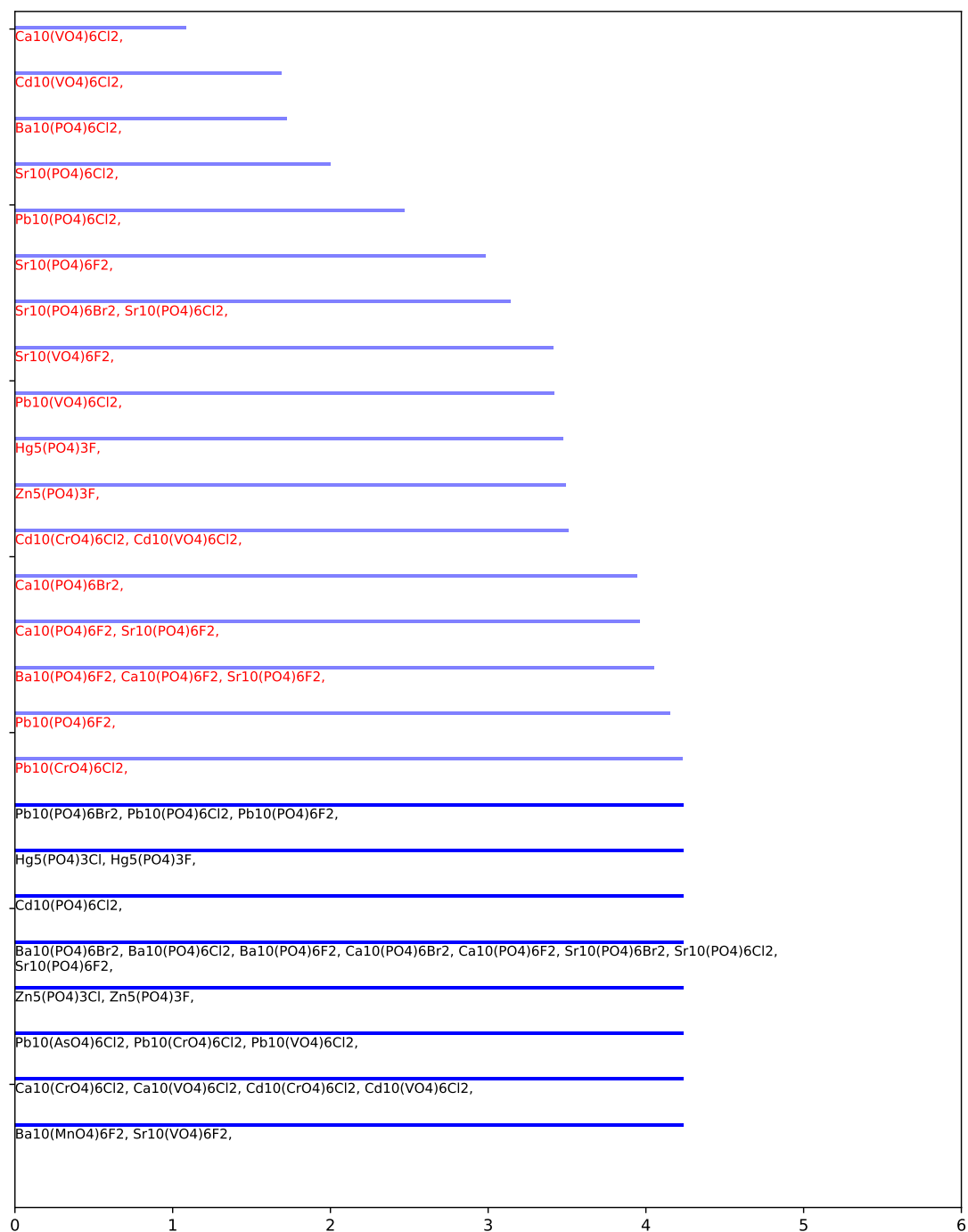

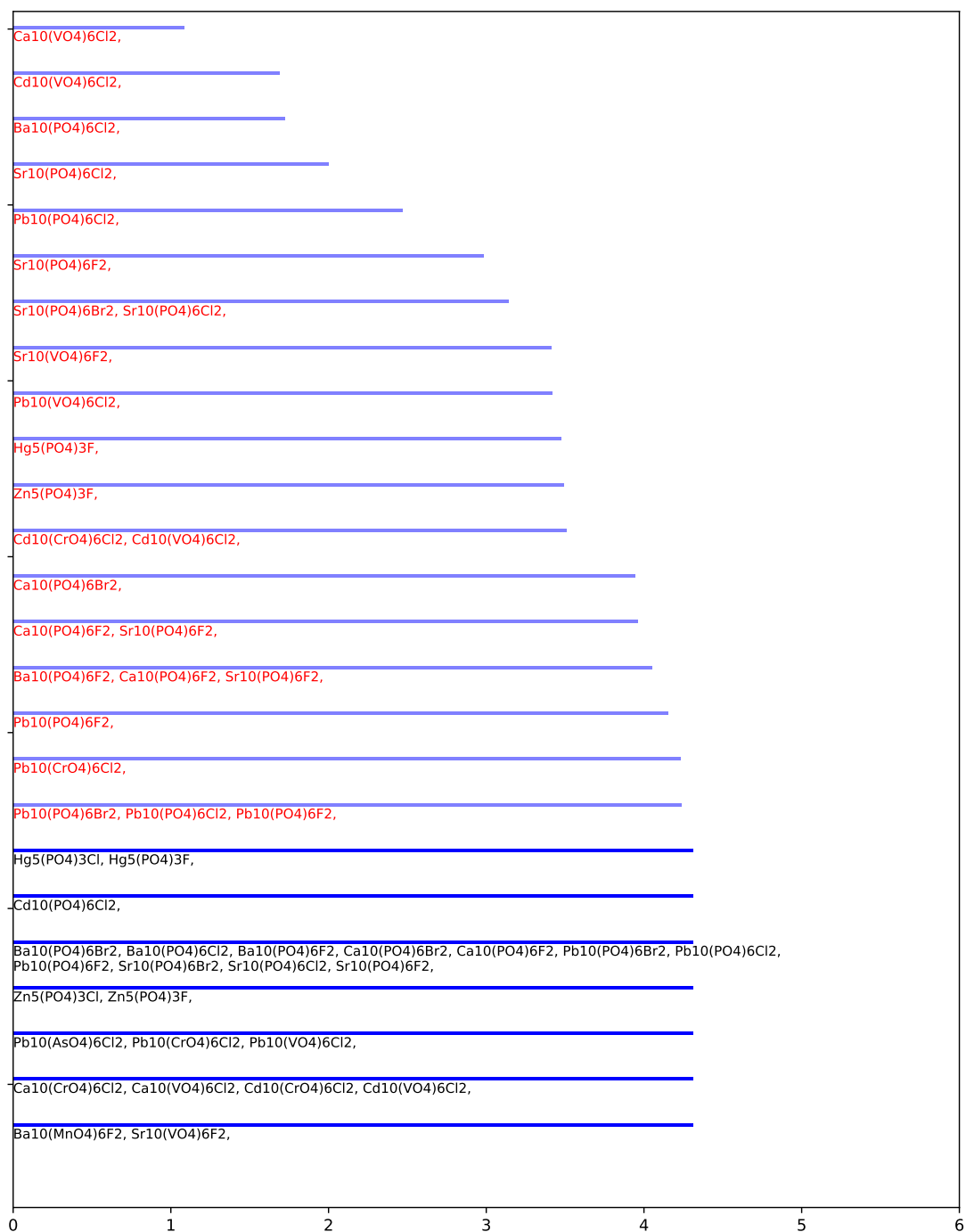

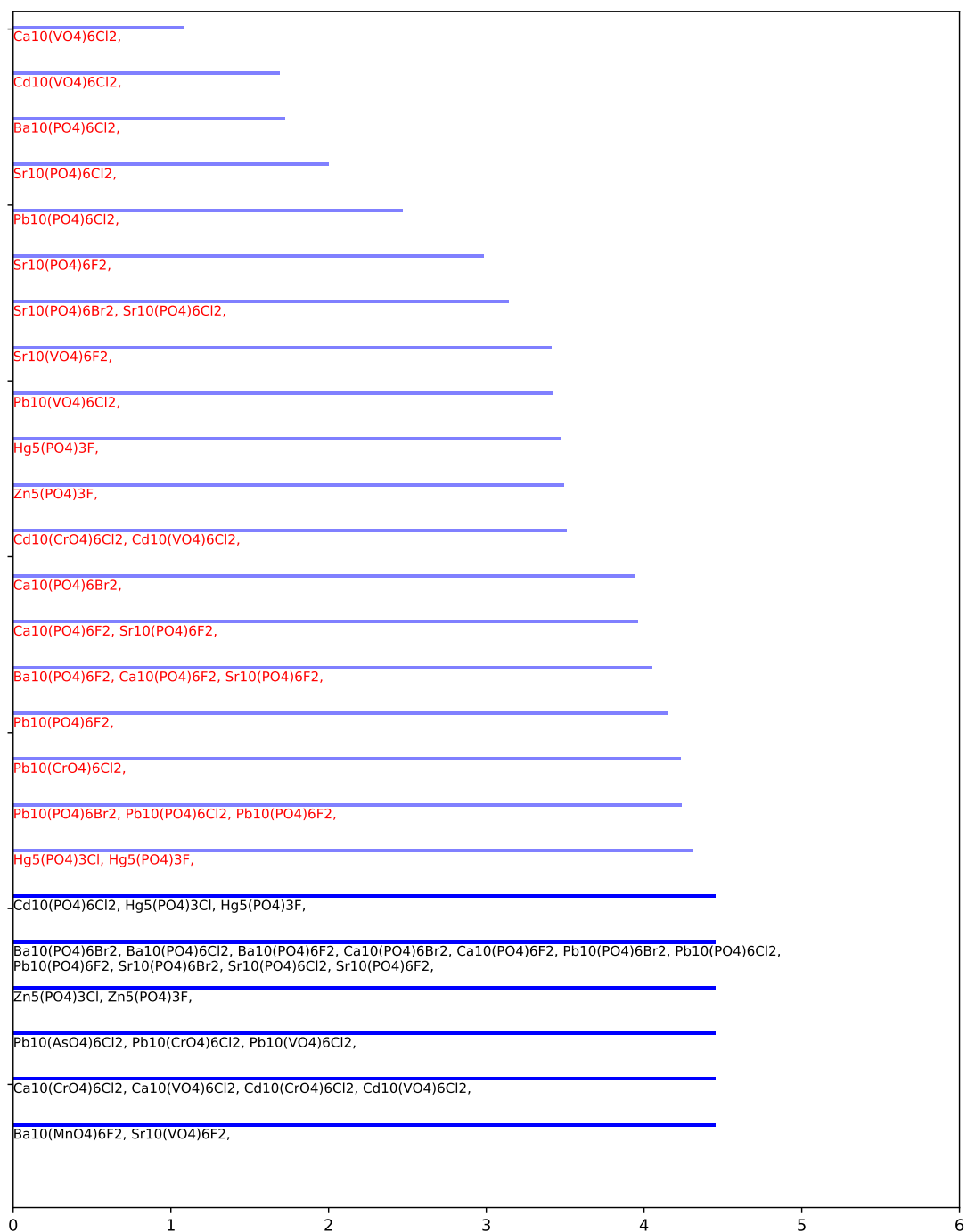

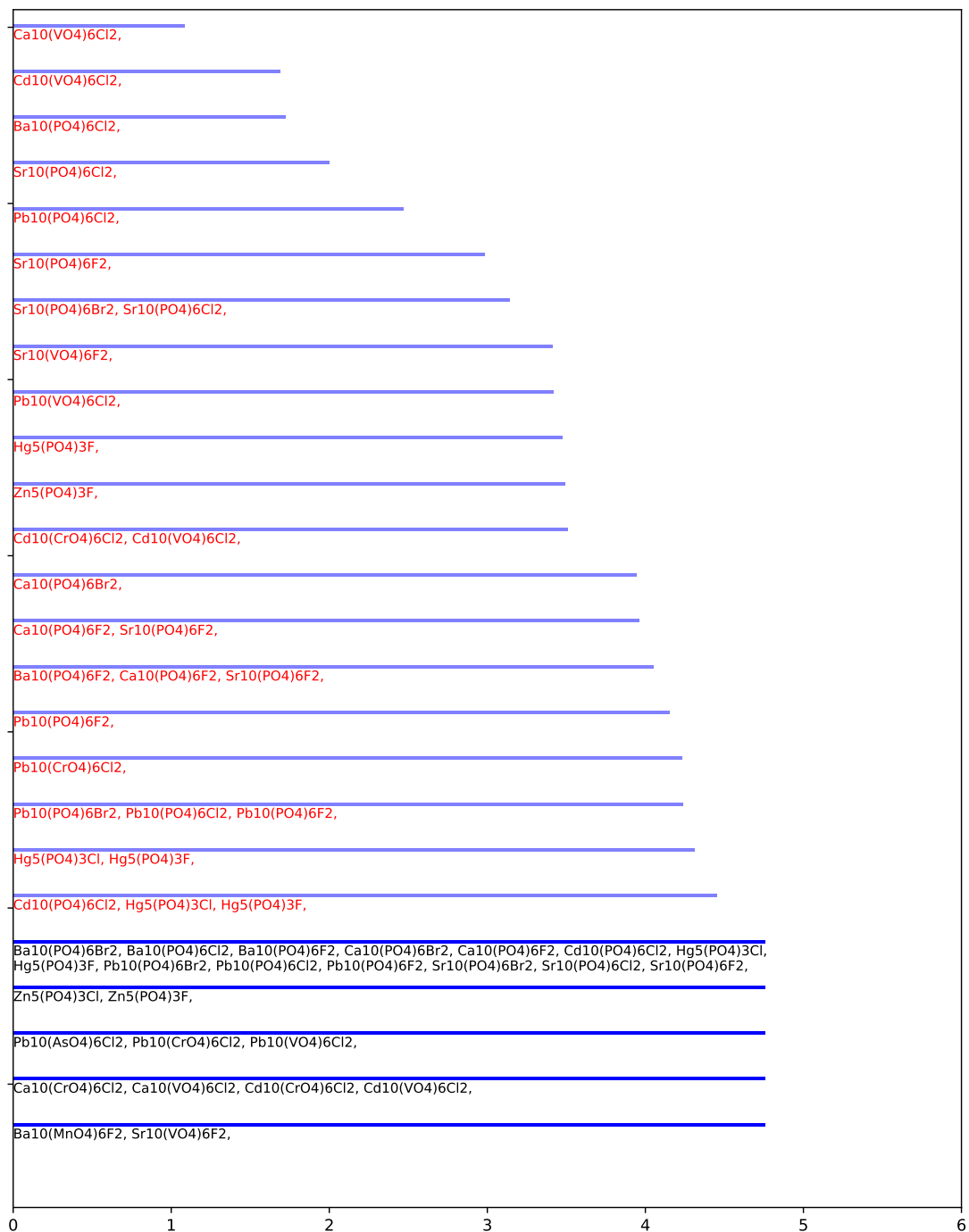

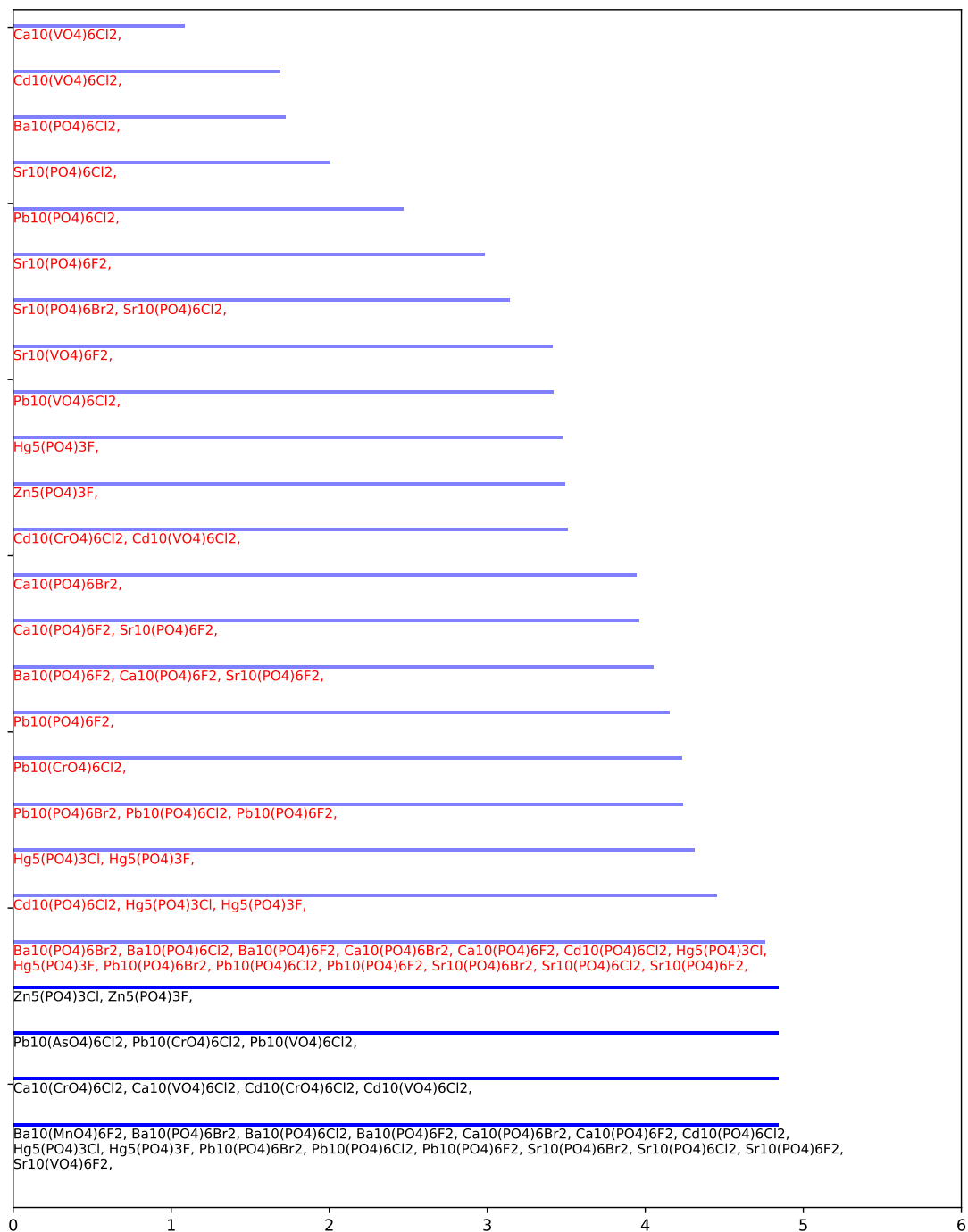

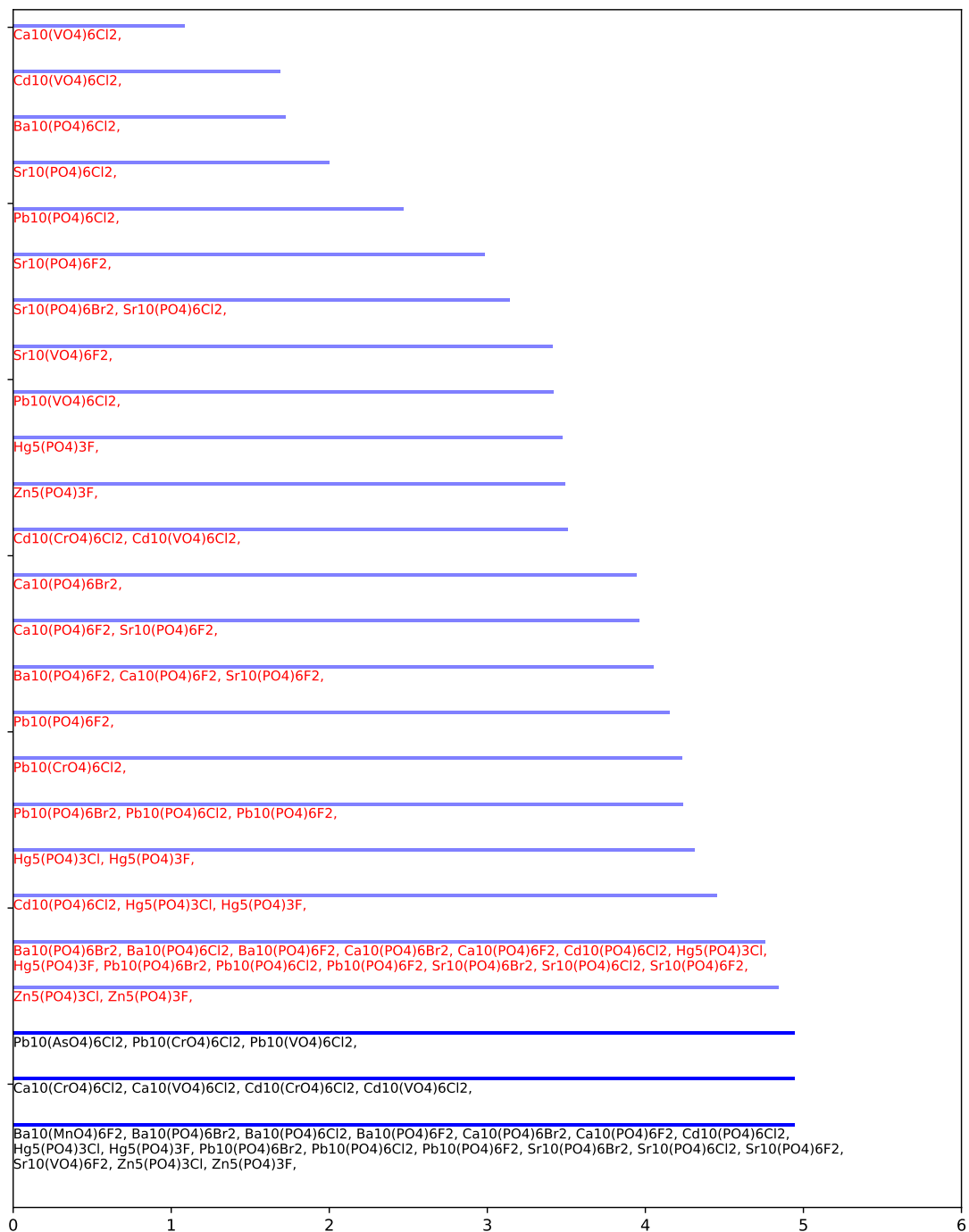

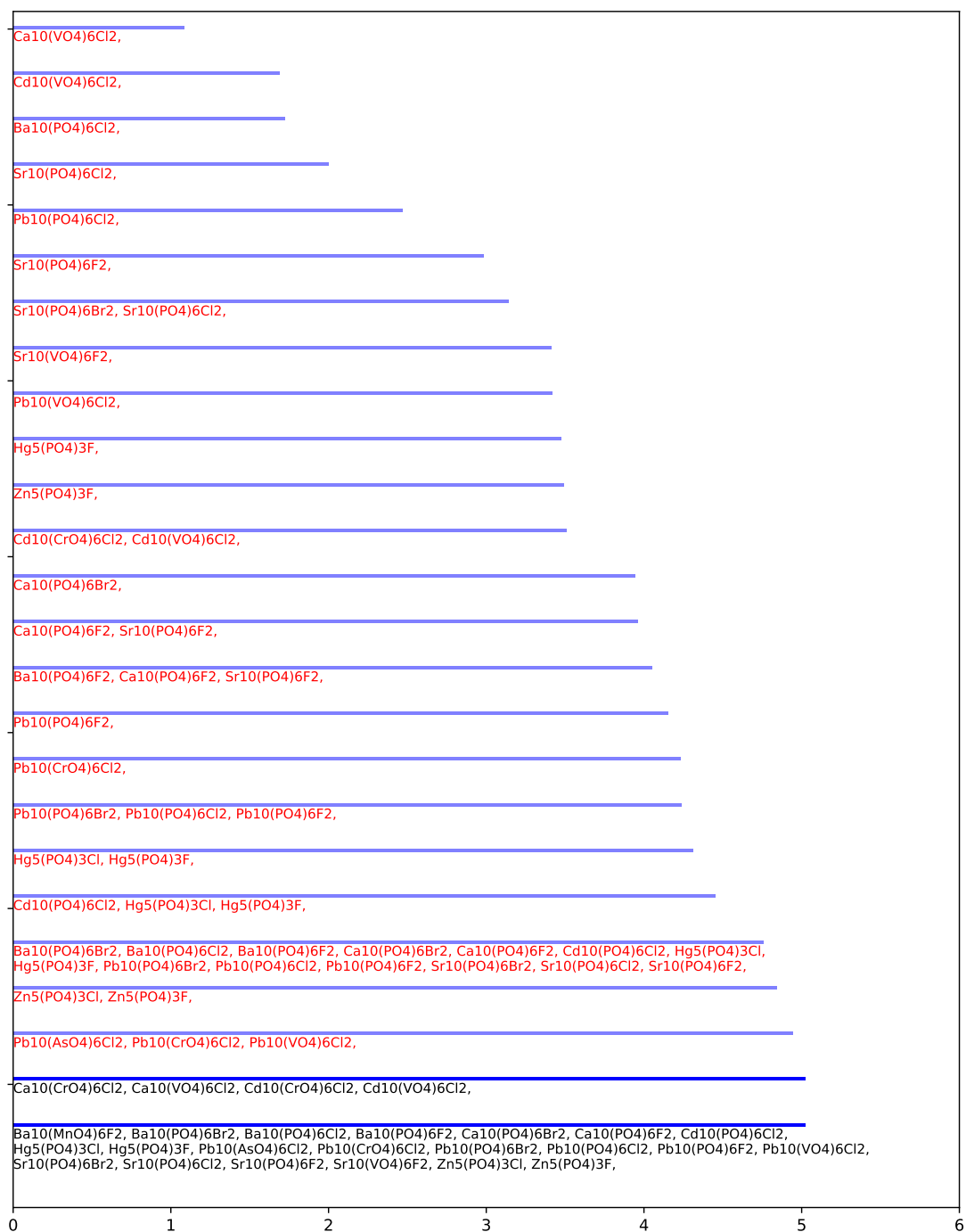

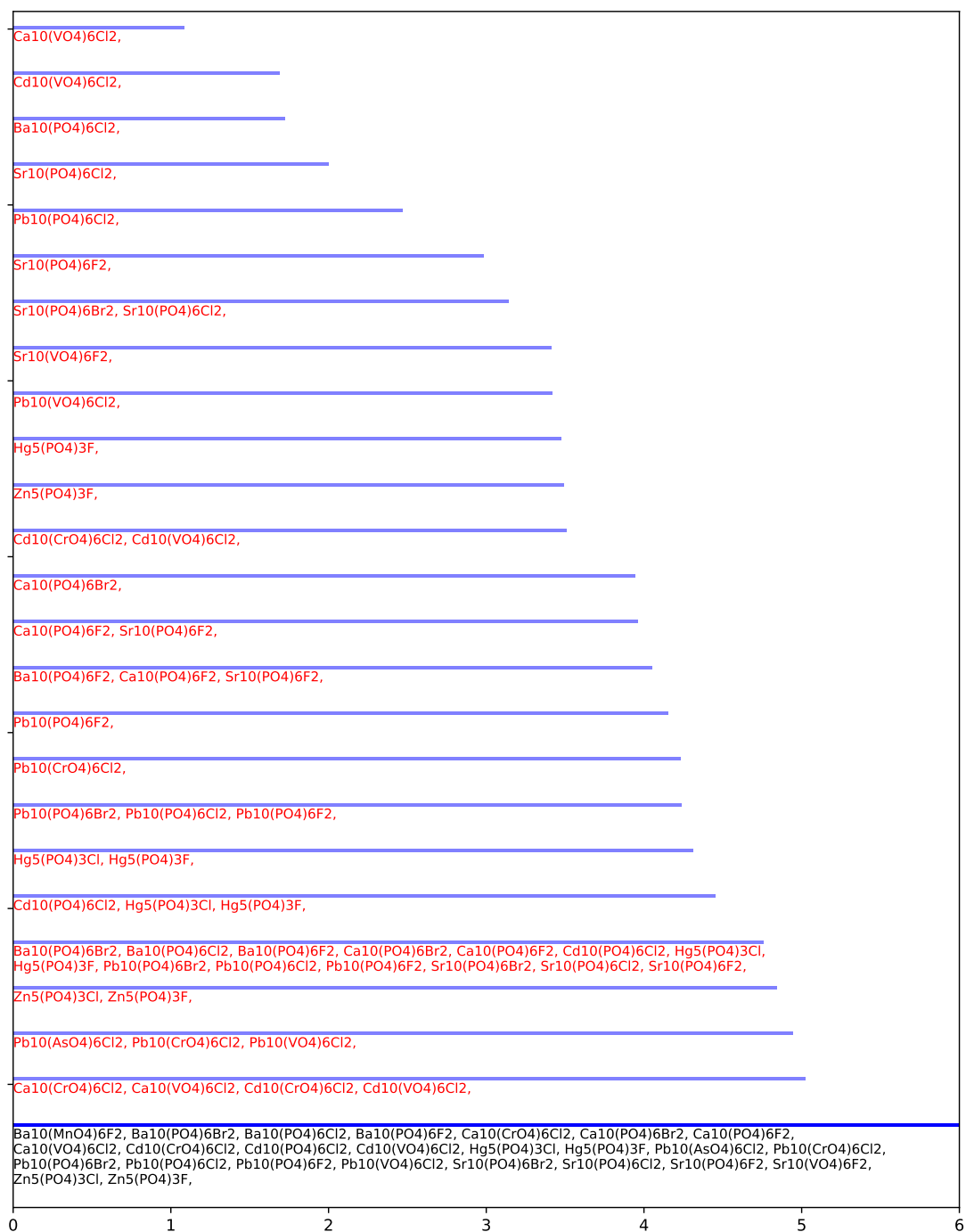

Supplement: Supplementary file 2 — Supplementary Information 2. [file 41598_2021_90070_MOESM2_ESM.pdf]

## Slide 1
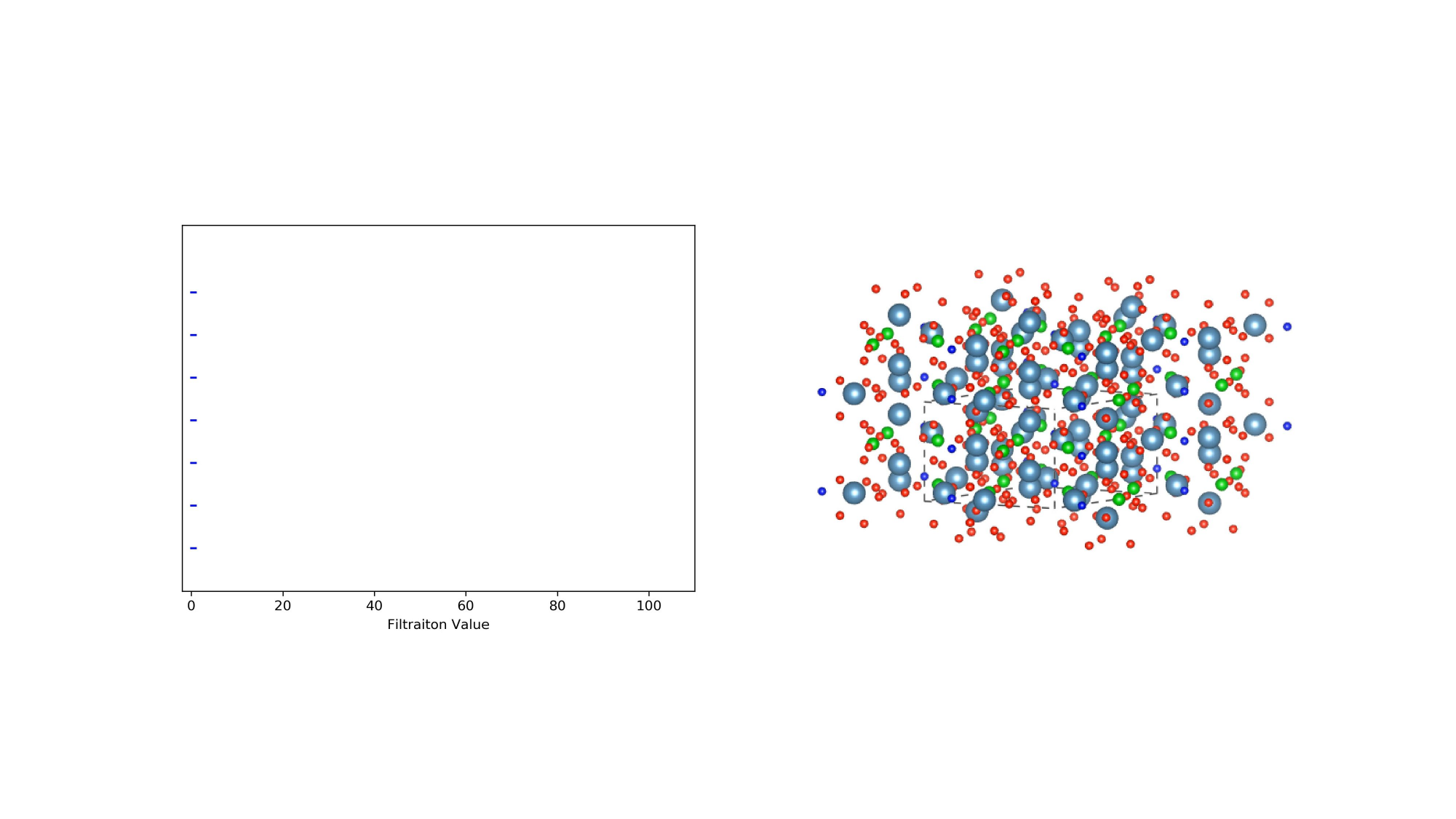

Supplement: Supplementary file 3 — Supplementary Video. [file 41598_2021_90070_MOESM3_ESM.pptx]
